# Mitochondrial-related genes as prognostic and metastatic markers in breast cancer: insights from comprehensive analysis and clinical models

**DOI:** 10.3389/fimmu.2024.1461489

**Published:** 2024-09-24

**Authors:** Yutong Fang, Qunchen Zhang, Cuiping Guo, Rongji Zheng, Bing Liu, Yongqu Zhang, Jundong Wu

**Affiliations:** ^1^ Department of Breast Surgery, Cancer Hospital of Shantou University Medical College, Shantou, Guangdong, China; ^2^ Department of Breast Surgery, Jiangmen Central Hospital, Jiangmen, Guangdong, China

**Keywords:** breast cancer, mitochondria, prognostic model, metastasis model, machine-learning

## Abstract

**Background:**

Breast cancer (BC) constitutes a significant peril to global women’s health. Contemporary research progressively suggests that mitochondrial dysfunction plays a pivotal role in both the inception and advancement of BC. However, investigations delving into the correlation between mitochondrial-related genes (MRGs) and the prognosis and metastasis of BC are still infrequent.

**Methods:**

Utilizing data from the TCGA database, we employed the “limma” R package for differential expression analysis. Subsequently, both univariate and multivariate Cox regression analyses were executed, alongside LASSO Cox regression analysis, to pinpoint prognostic MRGs and to further develop the prognostic model. External validation (GSE88770 merged GSE425680) and internal validation were further conducted. Our investigation delved into a broad spectrum of analyses that included functional enrichment, metabolic and immune characteristics, immunotherapy response prediction, intratumor heterogeneity (ITH), mutation, tumor mutational burden (TMB), microsatellite instability (MSI), cellular stemness, single-cell, and drug sensitivity analysis. We validated the protein and mRNA expressions of prognostic MRGs in tissues and cell lines through immunohistochemistry and qRT-PCR. Moreover, leveraging the GSE102484 dataset, we conducted differential gene expression analysis to identify MRGs related to metastasis, subsequently developing metastasis models via 10 distinct machine-learning algorithms and then selecting the best-performing model. The division between training and validation cohorts was set at 70% and 30%, respectively.

**Results:**

A prognostic model was constructed by 9 prognostic MRGs, which were DCTPP1, FEZ1, KMO, NME3, CCR7, ISOC2, STAR, COMTD1, and ESR2. Patients within the high-risk group experienced more adverse outcomes than their counterparts in the low-risk group. The ROC curves and constructed nomogram showed that the model exhibited an excellent ability to predict overall survival (OS) for patients and the risk score was identified as an independent prognostic factor. The functional enrichment analysis showed a strong correlation between metabolic progression and MRGs. Additional research revealed that the discrepancies in outcomes between the two risk categories may be attributed to a variety of metabolic and immune characteristics, as well as differences in intratumor heterogeneity (ITH), tumor mutational burden (TMB), and cancer stemness indices. ITH, TIDE, and IPS analyses suggested that patients possessing a low-risk score may exhibit enhanced responsiveness to immunotherapy. Additionally, distant metastasis models were established by PDK4, NRF1, DCAF8, CHPT1, MARS2 and NAMPT. Among these, the XGBoost model showed the best predicting ability.

**Conclusion:**

In conclusion, MRGs significantly influence the prognosis and metastasis of BC. The development of dual clinical prediction models offers crucial insights for tailored and precise therapeutic strategies, and paves the way for exploring new avenues in understanding the pathogenesis of BC.

## 1 Introduction

Breast cancer (BC), a prevalent malignancy among women, represents a significant global health challenge. Statistics show that around 2.3 million new cases of BC are reported annually, making up 11.7% of total cancer diagnoses. The annual mortality associated with breast cancer approaches 700,000, accounting for 6.9% of all fatalities linked to cancer (1). BC is a complicated and multifaceted process that is influenced by a combination of factors such as genetics, environment, and lifestyle. Furthermore, there has been a growing body of research in recent years showing a strong connection between tumor development and abnormal energy metabolism in tumors. In 2011, Weinberg et al. proposed the ten hallmarks of cancer, which include abnormal energy metabolism in tumor cells (2). Additionally, Warburg pioneered the theory that tumor cells exhibit a unique phenotype, characterized by increased glycolysis rates even under aerobic conditions (3). This metabolic behavior has been confirmed across various types of cancer, including BC (4). Tumor cells rely on mitochondrial oxidative phosphorylation (OXPHOS) for growth, as they can transition from aerobic glycolysis to OXPHOS for energy when glucose is scarce (5). Mitochondria, the quintessential powerhouses of eukaryotic cells, play a pivotal role in a myriad of cellular processes, encompassing metabolism, growth, differentiation, and apoptosis (6). Emerging evidence strongly suggests that mitochondrial dysfunction plays a pivotal role in the initiation and progression of cancer. A range of factors, including mitochondrial DNA abnormalities and defects in mitochondrial ribosomes, can disrupt the process of OXPHOS and compromise the function of the respiratory chain. The disruption leads to a lack of ATP synthesis, elevated calcium release, overproduction of reactive oxygen species (ROS), triggering of the mitochondrial unfolded protein response, and the alteration of multiple genes and signaling pathways related to the promotion or inhibition of cancer. Ultimately, these processes promote the occurrence and progression of cancers (7, 8). Furthermore, impaired mitochondrial function is linked to resistance to drugs and the survival of cancer stem cells (9, 10), making it a hot topic in the field of cancer research.

Recent research has increasingly focused on the role of mitochondrial-related genes (MRGs) function in the context of BC (11, 12). However, although these investigations offer crucial insights into the role of MRGs in BC, they also underscore the necessity for further research to comprehensively decipher the intricate biological mechanisms of mitochondria in BC, particularly their associations with metastasis and prognosis.

**Figure 1:**
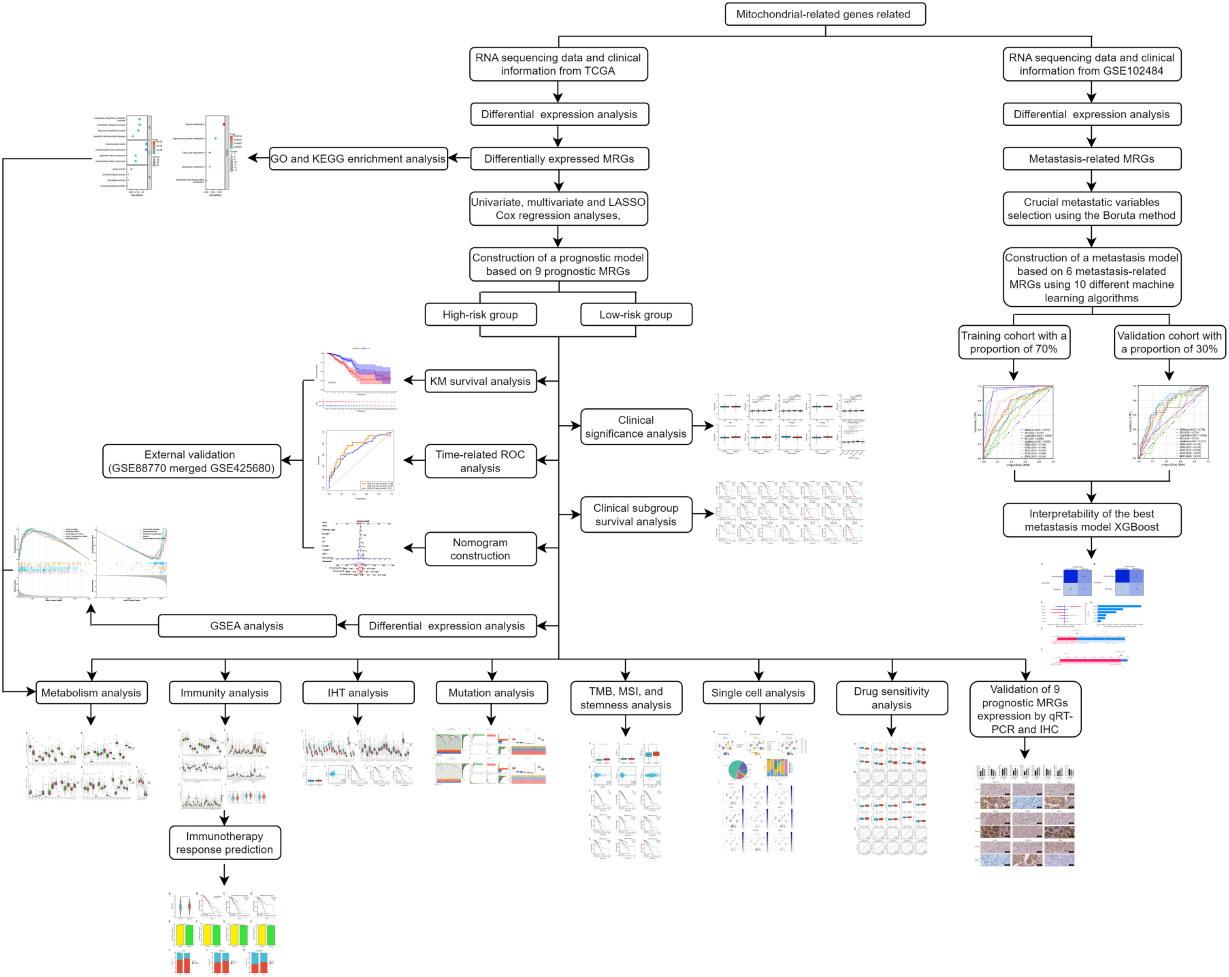
The flowchart graph depicts the methods and results in the present study.

In the present study, we constructed a novel mitochondrial-related risk model with the transcriptional information of BC samples from The Cancer Genome Atlas (TCGA) database to effectively predict the prognosis and immunotherapy responses of BC patients using 9 MRGs. Furthermore, to delve into the connection between MRGs and distant metastasis in BC, we developed diverse machine-learning models with 6 MRGs. The flow chart of the study is illustrated in **Figure 1**.

## 2 Methods

### 2.1 Data collection

We obtained level 3 RNA sequencing data (FPKM) for 1113 BC samples and 113 normal samples from the TCGA database (https://www.cancer.gov/tcga), along with their clinical details. Post exclusion of specimens lacking complete prognostic information, 1,055 BC samples were retained for analysis. We also integrated datasets with prognosis information from the Gene Expression Omnibus (GEO) database (https://www.ncbi.nlm.nih.gov/geo/) for external validation (GSE88770 merged with GSE42568). The metastasis model was constructed using the GSE102484 dataset, which included 101 metastatic and non-metastatic BC samples. In total, 2,030 MRGs were amassed from preceding studies as denoted in **Supplementary Table S1** (13).

### 2.2 Construction and validation of prognostic model based on MRGs

The differential gene expression analysis between normal and BC sample groups was performed with the “limma” R package, utilizing 2030 MRGs and applying the criteria of |Log fold change| >1 and adjusted P-value <0.05. Univariate and multivariate Cox regression analyses were performed to identify potential MRGs associated with OS. By constructing gene expression and survival object matrices, the Lasso-Cox regression model was fitted using the “glmnet” package. The optimal penalty parameter lambda was selected through ten-fold cross-validation, and the non-zero regression coefficients significantly associated with survival time were extracted. These coefficients reflect the contribution of each gene to the risk score. The risk score for each sample was determined by applying the formula: Risk score = (Coef1 * mRNA1 expression) + (Coef2 * mRNA2 expression) + … (Coefn * mRNAn expression), where “Coef” represents the coefficient derived from each mRNA’s LASSO regression analysis. Based on the above formula, we calculated the risk score for each BC sample and divided the BC samples into high-risk and low-risk groups according to the median value of the risk score. Kaplan-Meier analysis and log-rank test were used to compare overall survival (OS) in the two groups. We utilized the R packages “survival” and “timeROC” to conduct time-related ROC analysis to evaluate the prognostic efficacy of the model. Finally, the prognostic model was validated by an external validation cohort (GSE88770 merged with GSE425680). We also randomly selected 70% of the BC samples from TCGA as the internal validation cohort.

### 2.3 Nomogram construction and validation based on risk scores and clinical characteristics

We examined the correlation between the risk score and clinical characteristics and performed KM survival analysis for high-risk and low-risk groups across various clinical subgroups. Additionally, both univariate and multivariate Cox regression analyses were employed to ascertain the independent prognostic validity of the model, which incorporates the risk score and clinical characteristics. Subsequently, we developed a nomogram using the “rms” R package, incorporating risk score and clinical characteristics to predict the 1-, 3-, and 5-year OS probabilities for BC patients within the TCGA cohort. The precision of the nomogram was subsequently validated using the GEO cohort.

### 2.4 Functional enrichment analysis

Utilizing the “clusterProfiler” and “org.Hs.eg.db” R packages, we performed enrichment analyses on Gene Ontology (GO) and Kyoto Encyclopedia of Genes and Genomes (KEGG) based on the differentially-expressed MRGs. We applied an adjusted P-value threshold of less than 0.05 to discern the functional candidates. Furthermore, we executed a Gene Set Enrichment Analysis (GSEA) based on differentially-expressed genes between high-risk and low-risk groups, considering a P-value of less than 0.05 as statistically significant.

### 2.5 Metabolism analysis

Building on the findings from the functional enrichment analysis, we analyzed metabolism-related gene expression in two risk groups. From a study that was published (**Supplementary Table S2**) (14), we gathered 2752 metabolism-related genes, among which we constructed protein-protein interaction (PPI) networks for nucleotide, fatty acid, and amino acid metabolism-related genes and selected top 15 core genes for further analysis by Cytoscape (version 3.8.2).

### 2.6 Immunity analysis and immunotherapy response prediction

To explore the tumor immune landscape within two risk groups, we analyzed the expression of major histocompatibility complex (MHC) molecules, chemokines and receptors, immune cell infiltration, and evaluated the expression of prevalent immune checkpoint genes (ICGs) across two groups. Utilizing the single sample gene set enrichment (ssGSEA) algorithm (15), we quantified the abundance of 24 well-known immune cell types. Additionally, the CIBERSORT algorithm (16) enabled the evaluation of infiltration levels across 22 distinct immune cell populations. We further performed a comparative analysis of immune cell signatures between two risk groups, drawing upon data sourced from the TISIDB database (http://cis.hku.hk/TISIDB/download.php) (17). Moreover, the ESTIMATE algorithm, executed via the “estimate” R package, allowed us to calculate immune, stromal, and ESTIMATE scores, providing a comprehensive assessment of the tumor microenvironment.

The likelihood of each BC specimen from the TCGA database responding to immunotherapy was estimated using the Tumor Immune Dysfunction and Exclusion (TIDE) score (http://tide.dfci.harvard.edu). Additionally, the Immunophenoscore (IPS) algorithm, which leverages machine-learning to forecast responses to anti-CTLA4 and anti-PD-1 therapies (18), was applied to each BC sample for predictive insights. The IMvigor210 and GSE78220 cohorts were further used to validate the immunotherapy response predictive power of the model.

### 2.7 Intratumor heterogeneity analysis

ITH signifies the presence of varied cellular subpopulations within a single tumor, each distinguished by distinct genetic, phenotypic, or functional characteristics (19). We evaluated the ITH scores of each BC specimen utilizing the “DEPTH” package in R, classifying them into ITH-high and ITH-low cohorts based on the median ITH score. Subsequently, we conducted a differential gene expression analysis between two ITH groups utilizing the “limma” package in R, from which we identified the top 20 ITH-differentially-expressed genes for subsequent exploration of their expression in two risk groups.

### 2.8 Mutation, tumor mutational burden, microsatellite instability, cancer stemness, and single cell analysis

We conducted a comprehensive evaluation to ascertain disparities in mutations, TMB scores, MSI scores, and tumor stemness between two risk groups. Mutation details for BC specimens were sourced from the TCGA database, with the “maftools” R package utilized to generate waterfall plots that visually depict the mutational landscape for each risk group. Subsequently, we calculated the TMB score for individual samples. MSI scores were integrated from a previous study (20). Additionally, we applied a one-class logistic regression algorithm within the machine-learning domain (21) to derive the mRNA expression-based stemness index (mRNAsi) for each specimen. We separately categorized the samples according to the median values of TMB, MSI, and mRNAsi scores for subsequent analysis. We also conducted single-cell analyses on nine prognostic MRGs using data from the GSE148673 dataset, accessed via the TISCH web resource (http://tisch.comp-genomics.org) (22).

### 2.9 Drug sensitivity analysis

The R package “pRRophetic” was employed to determine the 50% inhibitory concentration (IC50) values of 236 medications for individuals using the Genomics of Drug Sensitivity in Cancer (GDSC) (https://www.cancerrxgene.org/) database (23). A Spearman correlation analysis was conducted on the risk score and IC50 values of the drugs, followed by the selection of the top 10 drugs with the most significant positive and negative correlations with the risk score for further examination. We compared the IC50 value of the drugs in two risk groups. Furthermore, we categorized samples into high-response and low-response groups based on the median cut-off value of the IC50 value for each drug. Additionally, we conducted a ROC analysis to evaluate the efficacy of the risk score in distinguishing between the high-response and low-response groups.

### 2.10 Construction of the metastasis model based on MRGs

We employed the GSE102484 dataset for the construction of the distant metastasis model. BC patients were categorized into distant metastasis and non-metastasis groups for subsequent analysis of differential gene expression between the two groups to identify metastasis-related MRGs based on the criteria of |Log fold change| >0.75 and adjusted P-value <0.05. Then we selected the metastasis-related MRGs for further selection of crucial metastatic variables using the Boruta method. The Boruta algorithm is a random forest (RF) based feature selection algorithm for identifying the most relevant features in a dataset. Its core steps include the construction of shadow features and voting in RF.

Then we used 10 different machine-learning algorithms, which were extreme gradient boosting (XGBoost), logistic regression (LR), light gradient boosting machine (LightGBM), RF, adaptive boosting (AdaBoost), gaussian naive bayes (GNB), complement naive bayes (CNB), multi-layer perceptron neural networks (MLP), support vector machine (SVM) and k-nearest neighbors (KNN), to construct the metastasis model with selected crucial metastatic variables. BC Patients from the GSE102484 dataset were allocated into training and validation cohorts with a proportion of 70% and 30%, respectively. The model’s performance was compared using the area under the curve (AUC), accuracy, sensitivity, specificity, positive predictive value (PPV), negative predictive value (NPV) and F1 value. The cross-validation method was used with a random seed set to 1 and the fold set to 10.

### 2.11 Interpretability of the metastasis model

Upon identifying the best-performing model, its classification efficacy was illustrated through a confusion matrix. Calibration curves were used to assess the consistency between the model’s predicted outcomes and actual results. Decision Curve Analysis (DCA) was performed to evaluate the practical value of the model in clinical settings. Moreover, the significance of each characteristic in the model was clarified by utilizing SHapley Additive exPlanations (SHAP) values obtained through the “shap” software package.

### 2.12 Statistical analysis

Statistical analyses were performed using the R software version 4.0.5 or Python Version 3.8. The Wilcoxon signed-rank test was used to compare the differences of continuous variables between two groups, and the Kruskal-Wallis test to more than two groups. Categorical variables were compared using the chi-square test. Correlation analysis was carried out with Spearman’s correlation analysis. A P-value of less than 0.05 was considered statistically.

### 2.13 Cell lines and quantitative real-time PCR

BC cell lines MCF-7 and MDA-MB-231, as well as the breast epithelial cell line MCF-10A were purchased from Procell (Wuhan, China). These were cultured by the manufacturer’s instructions. Total RNA was isolated from the cells using the RNAsimple total RNA kit from Tiangen in Beijing, China, according to the manufacturer’s instructions. Subsequently, we conducted a quantitative real-time polymerase chain reaction (qRT-PCR) with the PrimeScript™ RT reagent kit and the SYBR Premix Ex Taq™ II from Takara, Japan, following the instructions provided by the manufacturer. We chose GAPDH as the internal reference gene and determined relative expression levels utilizing the 2^-△△Ct^ method. **Supplementary Table S3** contains a list of the particular primers utilized in this research.

### 2.14 Immunohistochemistry

Tissue microarrays (F048Br01a) containing BC samples and adjacent non-tumor tissues were acquired from Bioaitech (Xian, China). For IHC staining, the slides underwent deparaffinization, rehydration, and antigen retrieval using a microwave. Antibodies were applied and incubated at 4°C overnight. The antibody concentrations are detailed in **Supplementary Table S4**. Secondary antibodies were then applied at room temperature for 30 minutes, followed by staining with DAB and counterstaining with hematoxylin. Two pathologists independently reassessed the IHC scores.

## 3 Result

### 3.1 Identification of prognostic MRGs

**Figure 2:**
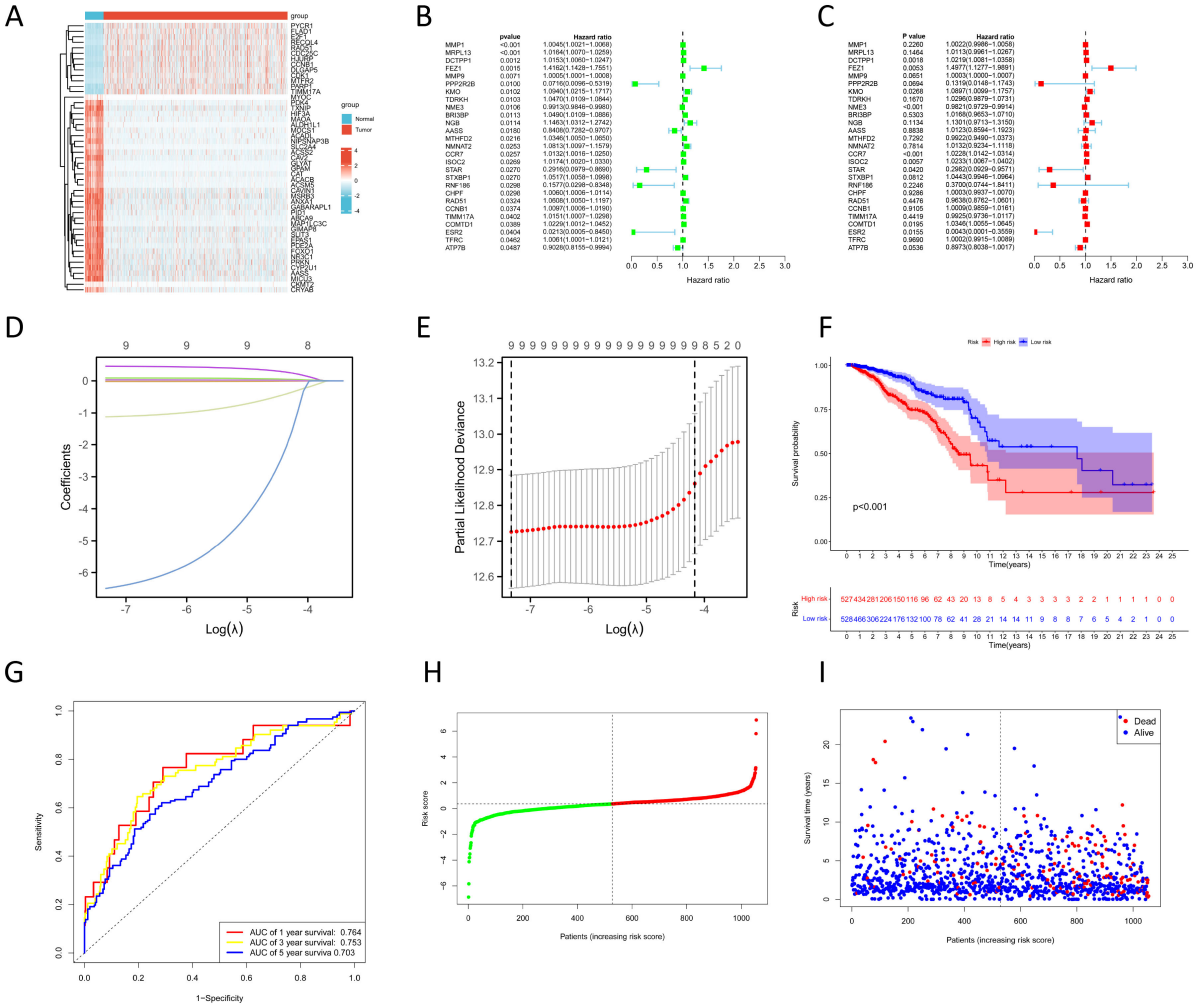
Construction of prognostic model based on MRGs. **(A)** Heat map shows differentially-expressed MRGs expression between normal and BC groups. **(B, C)** Univariate **(B)** and multivariate **(C)** Cox regression analyses to identify prognostic MRGs. **(D)** LASSO coefficient profiles. **(E)** Cross-validation for tuning parameter selection in the LASSO model. **(F)** KM survival curves to compare OS between high- and low-risk groups in the TCGA training cohort. **(G)** ROC curves for predicting 1-, 3-, and 5-year OS in the TCGA training cohort. **(H)** Distribution of risk scores between high- and low-risk groups in the TCGA training cohort. **(I)** Survival status of BC patients in the high- and low-risk groups in the TCGA training cohort.

The result of the differential gene expression analysis between normal and BC sample groups based on 2030 MRGs is shown in **Supplementary Table S5**, including a total of 365 differentially-expressed MRGs, and the heat map was plotted (**Figure 2A**). Subsequent univariate Cox regression analysis revealed that 27 differentially-expressed MRGs significantly associated with OS (p<0.05), depicted in **Figure 2B**. Progressing further, a multivariate Cox regression analysis was conducted on these 27 prognostic MRGs (**Figure 2C**), resulting in the selection of 9 critical MRGs for LASSO regression analysis to develop a prognostic model. The selected MRGs include DCTPP1, FEZ1, KMO, NME3, CCR7, ISOC2, STAR, COMTD1, and ESR2, with detailed information provided in **Table 1**.

**Table 1:** The information of 9 prognosis MRGs.

Gene	Full name	Location	Function of the encoded protein
DCTPP1	dCTP pyrophosphatase 1	16p11.2	DCTPP1 is an enzyme protein primarily involved in the metabolism of dCTP within cells. Through catalytic hydrolysis reactions, it breaks down dCTP into dCMP and pyrophosphate.
FEZ1	Fasciculation and elongation protein zeta 1	11q24.2	Protein encoded by FEZ1 primarily participates in neuronal development and synapse formation, exerting a crucial role within the nervous system.
KMO	Kynurenine 3-monooxygenase	1q43	KMO plays a crucial role in the kynurenine pathway, which is a metabolic pathway involved in the degradation of the amino acid tryptophan.
NME3	Nucleoside diphosphate kinase 3	16p13.3	NME3 is a protein that belongs to the nucleoside diphosphate kinase family. This family of enzymes plays a crucial role in cellular processes by catalyzing the transfer of phosphate groups between nucleoside diphosphates and nucleoside triphosphates, which are essential for energy transfer and cellular functions.
CCR7	C-C chemokine receptor 7	17q21.2	CCR7 is a protein that belongs to the G-protein coupled receptor family and is involved in mediating cell migration and immune responses. It is specifically known for its role in guiding immune cells to lymph nodes and other secondary lymphoid organs, where immune responses are orchestrated.
ISOC2	Isochorismatase domain containing 2	19q13.42	ISOC2 is a protein that possesses an isochorismatase-like domain, which suggests its potential involvement in certain enzymatic activities related to metabolic processes.
STAR	Steroidogenic acute regulatory protein	8p11.23	STAR is a crucial regulatory protein involved in the synthesis of steroid hormones, which are essential for various physiological processes in the body. The STAR protein facilitates the transport of cholesterol, a precursor molecule, into the mitochondria of steroid-producing cells, where it serves as the building block for steroid hormone production.
COMTD1	Catechol-O-methyltransferase containing domain 1	10q22.2	COMTD1 is a protein that contains a domain similar to catechol-O-methyltransferase, an enzyme involved in the metabolism of catechol compounds, including neurotransmitters like dopamine and catecholamines.
ESR2	Estrogen receptor 2	14q23.2-q23.3	ESR2 refers to Estrogen Receptor β, which is a specific protein in the human body. ESR2 is essential for the functioning of the reproductive system and has effects throughout the body.

Additionally, Kaplan-Meier (KM) survival analyses were performed to examine the differences in OS between high- and low-expression groups of these 9 prognostic MRGs, categorized by their median expression levels. Illustrated in **Supplementary Figure S1**, our findings revealed that high expression of DCTPP1 and low expression of CCR7 were correlated with reduced OS in BC patients (p<0.05).

### 3.2 Construction and validation of prognostic model

The LASSO regression and its cross-validation are illustrated in **Figures 2D** and **E**, respectively. The model calculates the risk score for each sample using the formula: Risk score = (0.021 * DCTPP1) + (0.434 * FEZ1) + (0.085 * KMO) + (-0.017 * NME3) + (0.021 * CCR7) + (0.023 * ISOC2) + (-0.974 * STAR) + (0.033 * COMTD1) + (-5.701 * ESR2). This formula stratified BC samples into high-risk and low-risk groups based on the median risk score. A heat map was plotted to compare 9 prognostic MRGs expression between high- and low-risk groups (**Supplementary Figure S2A**). KM survival analysis demonstrated significantly poorer OS in the high-risk group compared to the low-risk group (p<0.001) (**Figure 2F**). To assess the model’s predictive accuracy for 1-, 3-, and 5-year OS, time-dependent ROC analysis was performed, yielding AUC values of 0.764, 0.753, and 0.703 respectively (**Figure 2G**). **Figures 2H** and **I** display the risk score distribution and survival status across the low- and high-risk groups, highlighting a correlation between elevated risk score and increased mortality in BC patients.

**Figure 3:**
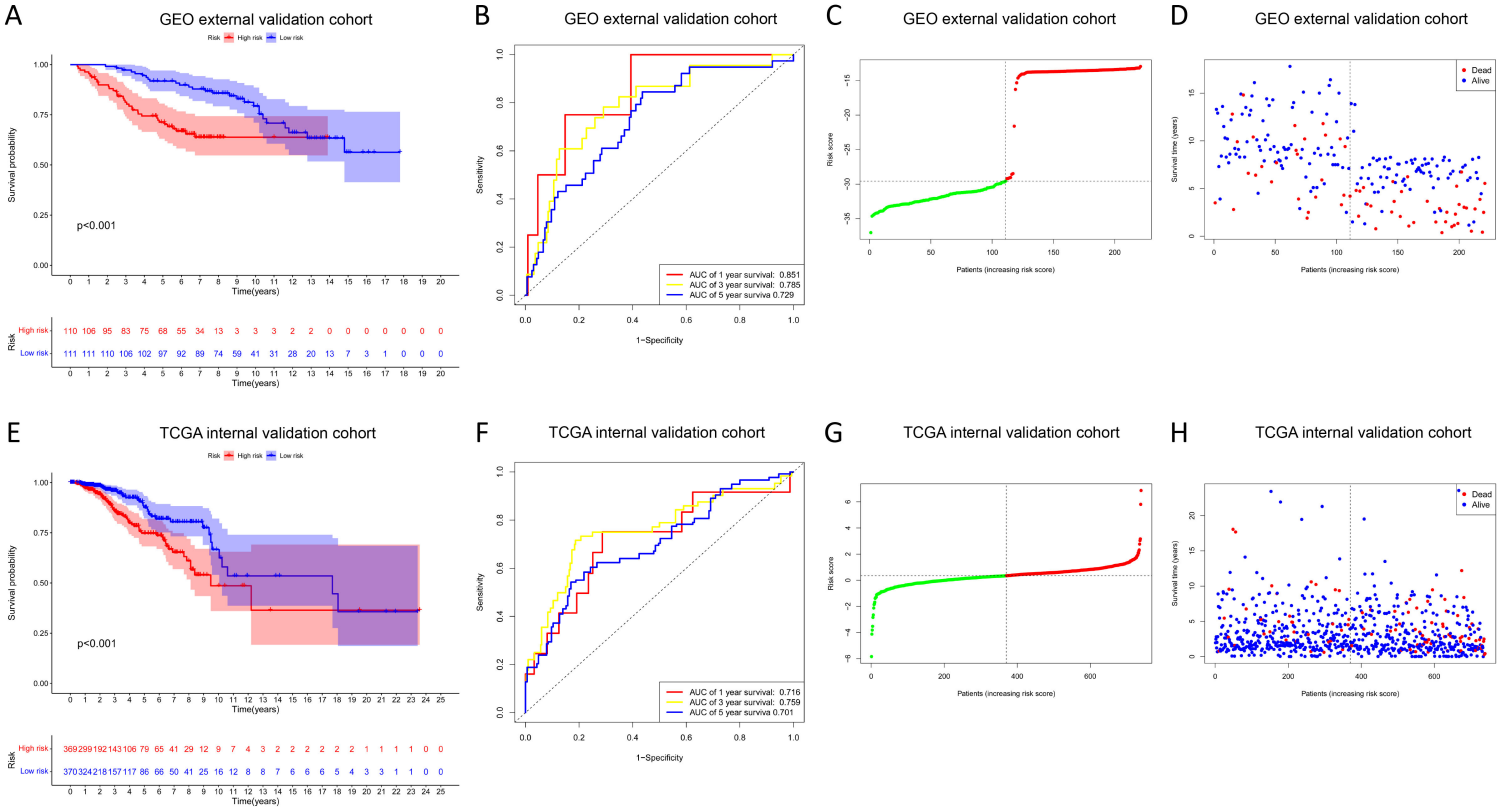
Validation of the prognostic model. **(A, E)** KM survival curves show the OS between high- and low-risk groups in the GEO external validation **(A)** and TCGA internal validation cohort **(E)**. **(B, F)** ROC curves for predicting 1-, 3-, and 5-year OS in the GEO external validation **(B)** and TCGA internal validation cohort **(F)**. **(C, G)** Distribution of risk scores between high- and low-risk groups in the GEO external validation **(C)** and TCGA internal validation cohort **(G)**. **(D, H)** Survival status of BC patients in the high- and low-risk groups in the GEO external validation **(D)** and TCGA internal validation cohort **(H)**.

In the GEO external validation cohort (GSE88770 merged with GSE425680), patients in the high-risk group had worse OS (**Figure 3A**), which was consistent with the TCGA training cohort. The AUC of the time-dependent ROC curves for predicting 1-, 3-, and 5-year OS were 0.851, 0.785, and 0.729, respectively (**Figure 3B**). The correlation between increasing risk score and higher mortality rate in patients was further substantiated (**Figures 3C, D**). Clinical characteristics of BC patients from the GEO validation cohort are shown in **Supplementary Table S6**. For internal validation, a subset comprising 70% of BC samples from the TCGA database was randomly selected, and the above results were also validated (**Figures 3E-H**).

In our study, 6 MRGs showed a marked increase in expression in BC tissues, while 3 MRGs exhibited a decrease compared to normal tissues (p<0.001) (**Supplementary Figure S2B**). As revealed by ROC analysis, DCTPP1 showed the best diagnostic performance for BC among 9 MRGs, achieving an AUC of 0.920, sensitivity of 0.803, and specificity of 0.930 (**Supplementary Figure S2C**). Furthermore, FEZ1 (AUC=0.917), NME3 (AUC=0.833), ISOC2 (AUC=0.850), COMTD1 (AUC=0.842), and ESR2 (AUC=0.904) also demonstrated substantial diagnostic capabilities. The differential expression of these 9 MRGs was further corroborated in BC cell lines compared to a normal breast epithelial cell line (**Supplementary Figure S2D**). To confirm these findings at the protein level, we conducted IHC on both normal and BC tissues. Representative IHC images are presented in **Supplementary Figure S2E**.

### 3.3 Clinical significance and clinical subgroup survival analysis of the risk groups

Clinical characteristics of BC patients from TCGA are shown in **Table 2**. We analyzed the clinical significance of the risk score and found that it is significantly associated with T stage, estrogen receptor (ER) status, progesterone receptor (PR) status, human epidermal growth factor receptor 2 (HER2) status, survival status, and PAM50 subtype (p<0.01) (**Supplementary Figure S3**). Notably, the risk score were notably elevated in T4 stage cases, suggesting the involvement of MRGs in the invasion of the skin and chest wall in locally advanced BC. Furthermore, negative ER and PR status, positive HER2 status, and deceased survival status were associated with higher risk score. By analyzing the relationship between the risk score and PAM50 breast cancer subtypes, we found that the risk score is significantly higher in Luminal B, HER2-enriched, and Basal-like subtypes compared to Normal-like and Luminal A subtypes. Additionally, as illustrated in **Supplementary Figure S4**, 9 MRGs exhibited significant correlations with various clinical characteristics.

**Table 2:** Clinical characteristics of BC patients from TCGA.

Clinical characteristics	Group	No. of case (%)
Age (year)	<60	588 (53.73)
	≥60	467 (46.27) 275 (26.07) 610 (57.82)
T stage	T1	
	T2	
	T3	134 (12.70)
	T4	33 (3.13)
	Unknown	3 (0.28)
N stage	N0	499 (47.30)
	N1	347 (32.89)
	N2	116 (11.0)
	N3	74 (7.01)
	Unknown	19 (1.80) 879 (83.32)
M stage	M0	
	M1	20 (1.90)
	Unknown	156 (14.79)
Pathologic stage	I	180 (17.06)
	II	597 (56.59)
	III	236 (22.37)
	IV	18 (1.71)
	Unknown	24 (2.27) 770 (72.99)
ER status	Positive	
	Negative	237 (22.46)
	Unknown	48 (4.55)
PR status	Positive	670 (63.51)
	Negative	334 (31.66)
	Unknown	51 (4.83)
HER2 status	Positive	153 (14.50) 544 (51.56)
	Negative	
	Unknown	358 (33.93)
Survival status	Alive	908 (86.07)
	Dead	147 (13.93)

In addition, we analyzed the prognostic differences between high- and low- risk groups in different clinical subgroups. As depicted in **Supplementary Figure S5**, in the majority of subgroups, except for the HER2-positive, Normal-like, Luminal A, and Basal-like subgroups, the OS for high-risk patients is notably shorter compared to that of low-risk patients (p<0.05). These findings highlight the clinical applicability and reliability of the prognostic model.

### 3.4 Construction and validation of nomogram

**Figure 4:**
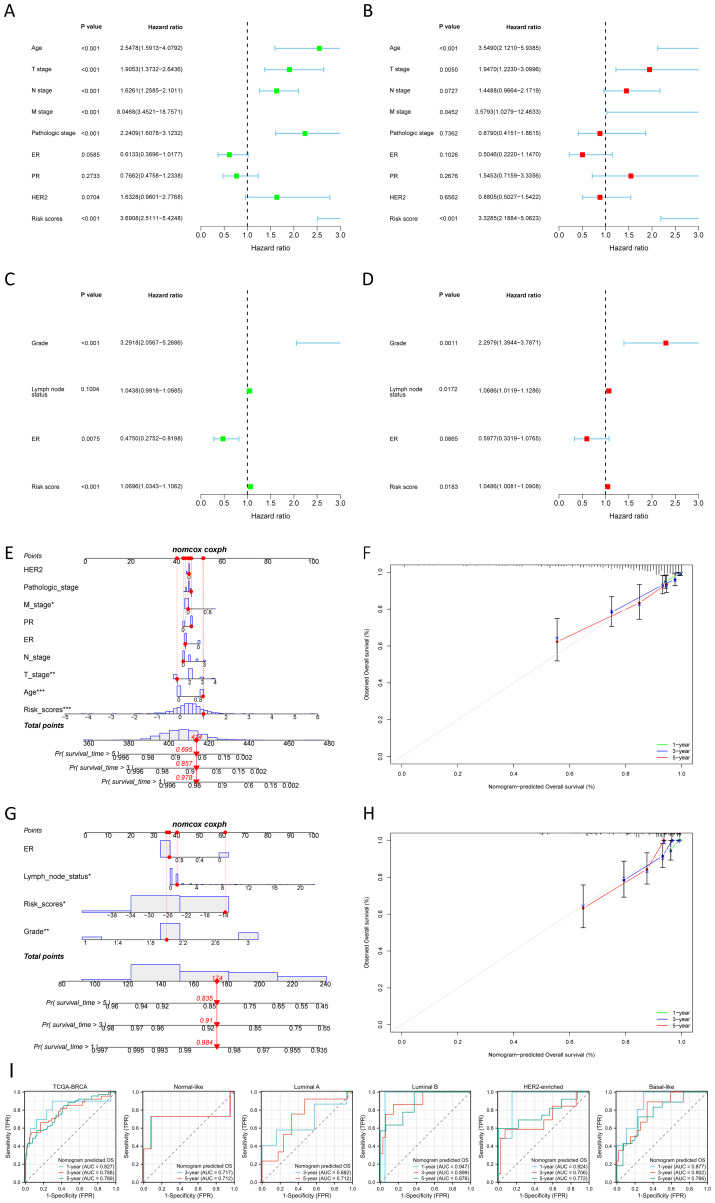
Construction and validation of nomogram. **(A, B)** Univariate **(A)** and multivariate **(B)** Cox regression analysis of the risk score and clinical characteristics in the TCGA training cohort. **(C, D)** Univariate **(C)** and multivariate **(D)** Cox regression analysis of the risk score and clinical characteristics in the GEO validation cohort. **(E, F)** The nomogram **(E)** for predicting the 1-, 3- and 5-year OS probabilities, and calibration curves **(F)** of the nomogram to predict 1-, 3- and 5-year OS probabilities in the TCGA training cohort. **(G, H)** The nomogram **(G)** for predicting the 1-, 3-and 5-year OS probabilities, and calibration curves **(H)** of the nomogram to predict 1-, 3- and 5-year OS probabilities in the GEO validation cohort. **(I)** Nomogram predicted OS in all BC samples and each PAM50 subtype. *P < 0.05, **P < 0.01, ***P < 0.001.

The nomogram, constructed using multifactorial regression analysis, forecasts patient survival by representing various clinical indicators as a series of distinct line segments on a two-dimensional Cartesian coordinate system. In univariate and multivariate Cox regression analysis, we combined the OS of BC patients with their clinical characteristics to determine whether the risk score from the prognostic model is an independent predictor of survival. In the TCGA training cohort, univariate Cox regression analysis revealed that age, T stage, N stage, M stage, pathologic stage, and risk score were significantly associated with OS (p<0.001) (**Figure 4A**). The results of the multivariate Cox regression analysis suggested that both risk score and age were independent predictors for OS in BC patients (p<0.001) (**Figure 4B**). We also confirmed that the risk score was an independent prognostic indicator in the GEO validation cohort (GSE88770 merged with GSE425680) through univariate and multivariate Cox regression analysis (**Figures 4C, D**). To improve the clinical applicability of the constructed prognostic model, we developed a nomogram incorporating age, T stage, N stage, M stage, pathologic stage, ER status, PR status, HER2 status, and risk score. This nomogram was utilized to predict the 1-, 3-, and 5-year OS probabilities of BC patients in the TCGA training cohort (**Figure 4E**). The calibration curve further substantiated the consistency between the actual OS of patients and the predictions made by the nomogram (**Figure 4F**). We also constructed a nomogram in the GEO validation cohort based on ER status, lymph node status, grade, and risk score (**Figure 4G**), with the calibration curve displayed in **Figure 4H**. We further evaluated the predictive performance of the nomogram for OS across all BC types and within each PAM50 subtype by plotting time-dependent ROC curves. As illustrated in **Figure 4I**, the AUCs for predicting 1-year, 3-year, and 5-year OS in all BC patients from TCGA were 0.827, 0.788, and 0.789, respectively. Among the various subtypes, the nomogram exhibited moderate predictive accuracy for OS in Normal-like and Luminal A BC patients, but demonstrated superior predictive performance in Luminal B, HER2-enriched, and Basal-like subtypes. Collectively, these results suggested that the nomogram, which combined the risk score with other clinical characteristics, exhibited superior prognostic predictive performance.

### 3.5 Functional enrichment analysis

**Figure 5:**
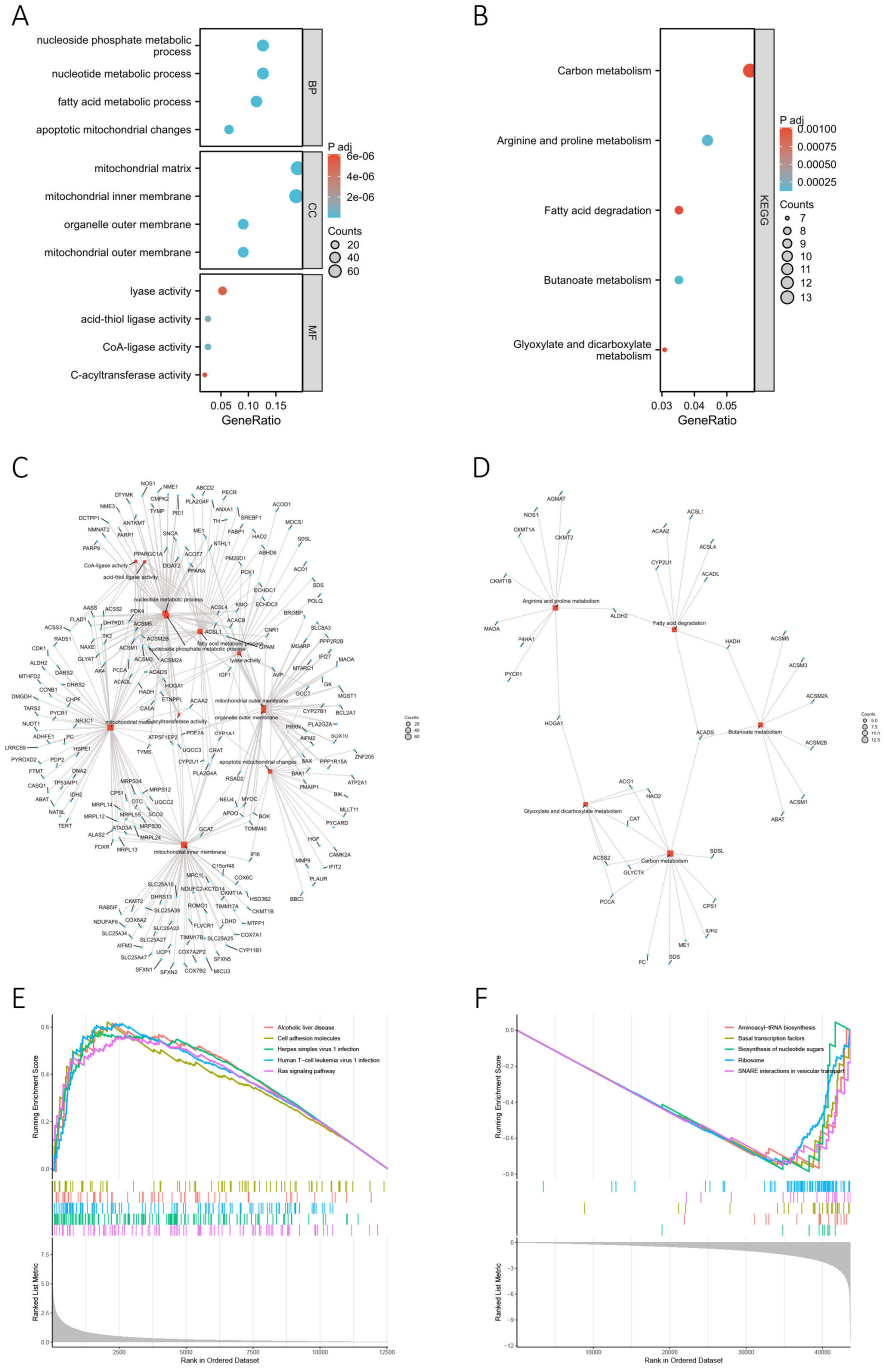
Functional enrichment analysis. **(A, B)** The bubble chart presents the results of the GO **(A)** and KEGG **(B)** enrichment analyses based on the differentially-expressed MRGs. **(C, D)** The network diagram shows the results of the GO **(C)** and KEGG **(D)** enrichment analyses. Blue nodes (circular) represent molecules, red nodes (square) represent categories, and the lines indicate the relationships between entries and molecules. **(E, F)** GSEA analysis based on differentially-expressed genes between high-risk **(E)** and low-risk groups **(F)**.

We conducted GO and KEGG enrichment analyses based on 356 differentially-expressed MRGs to investigate the potential biological functions of MRGs in the progression of BC. The corresponding results are presented in **Supplementary Tables S7** and **S8**, respectively. The key findings of the GO enrichment analysis are illustrated in the bubble chart (**Figure 5A**) and network diagram (**Figure 5C**). These results suggested that the genes annotated to biological processes (BP) were primarily associated with metabolic processes, particularly those involving nucleotides, nucleoside phosphates, and fatty acid. The cellular component (CC) primarily comprised the mitochondrial matrix, inner membrane, and outer membrane, while the molecular function (MF) was characterized by lyase activity, acid-thiol ligase activity, CoA-ligase activity, and C-acyltransferase activity. The key outcome of the KEGG enrichment analysis is depicted in **Figures 5B** and **D**, indicating the involvement of differentially-expressed MRGs in metabolic processes such as carbon metabolism, fatty acid metabolism, and amino acid metabolism. Based on these findings, we concluded that MRGs might play a role in regulating the progression of metabolism during the development of BC. Additionally, GSEA analysis conducted on differentially-expressed genes between high-risk and low-risk groups uncovered a significant association between the risk score and various pathways in the high-risk group (**Figure 5E**). These pathways included alcoholic liver disease, cell adhesion molecules, herpes simplex virus 1 infection, human T-cell leukemia virus 1 infection, and the Ras signaling pathway. In the low-risk group, pathway enrichment primarily centered around aminoacyl-tRNA biosynthesis, basal transcription factors, biosynthesis of nucleotide sugars, ribosome, and SNARE interactions in vesicular transport (**Figure 5F**). The detailed GSEA results for the high-risk and low-risk groups can be found in **Supplementary Tables S9** and **S10**, highlighting a robust correlation between the risk score and biological processes crucial to the development of BC, such as cell proliferation, differentiation, cell migration, immune regulation, biosynthesis, and gene transcription.

### 3.6 Metabolism analysis

**Figure 6:**
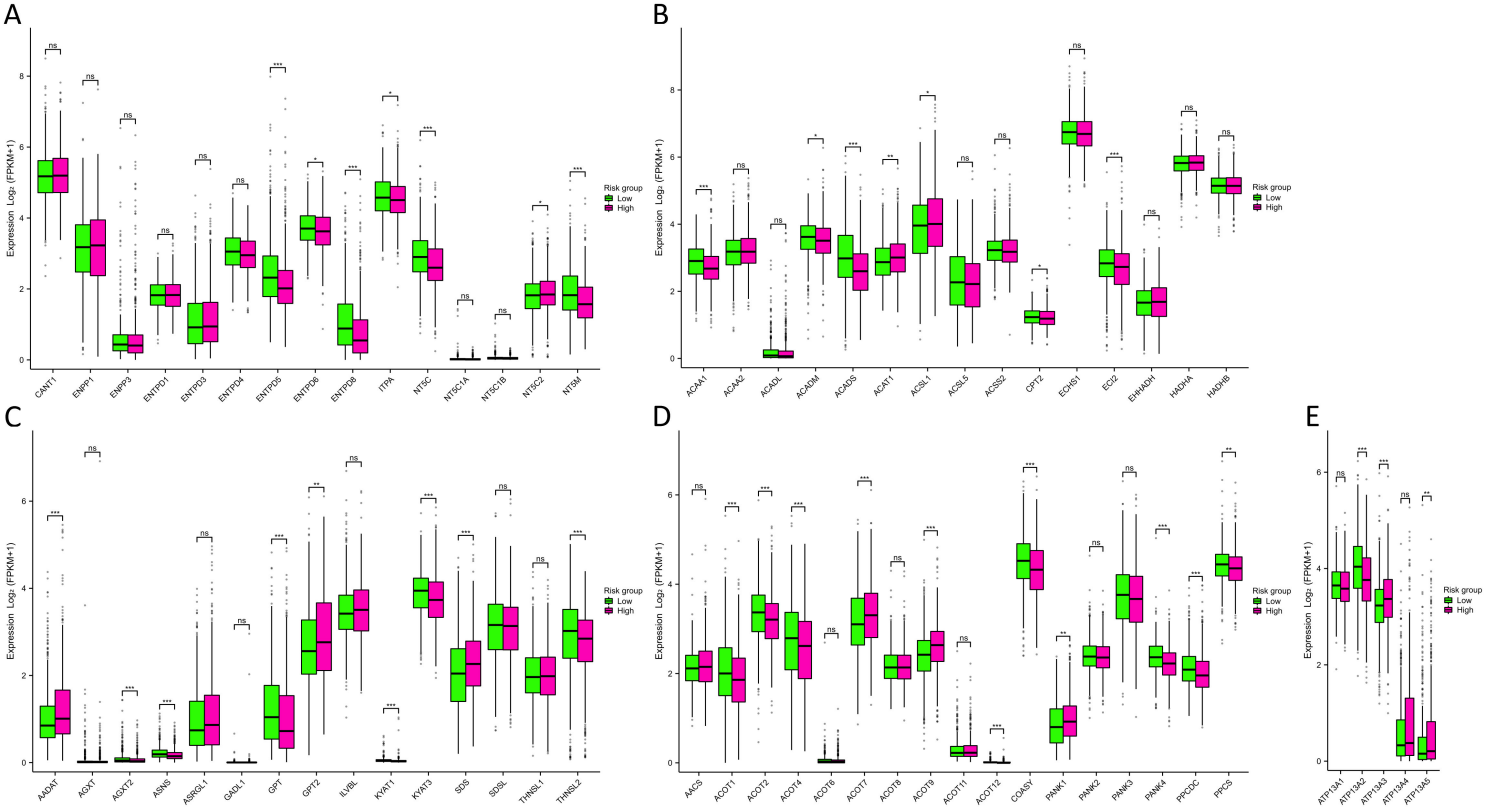
Metabolism analysis between two risk groups. **(A-E)** Differences in nucleotide **(A)**, fatty acid **(B)**, amino acid **(C)**, CoA **(D)**, and ATPase **(E)** metabolism-related genes expression between two risk groups. NS indicates no statistical difference, *P < 0.05, **P < 0.01, ***P < 0.001.

The outcomes of the functional enrichment analysis revealed that MRGs were primarily associated with the progression of metabolism. To investigate the metabolic characteristics between two risk groups, we compared metabolism-related genes expression between two risk groups, including 63 nucleotide, 139 fatty acid, 38 amino acid, 17 CoA, and 5 ATPase metabolism-related genes (**Supplementary Table S2**). Subsequently, we constructed PPI networks for 63 nucleotide, 139 fatty acid, and 38 amino acid metabolism-related genes. We identified 15 hub genes from each PPI network for further analysis (**Supplementary Figure S6**). From the results (**Figure 6A**) we found that in the high-risk group, the enzymes catalyzing nucleotide hydrolysis were down-expressed, such as NTPD5, NTPD8, NT5C and NT5M (p<0.001), revealing a function role in maintaining the rapid proliferation of tumor cells by inhibiting nucleotide hydrolysis. We also observed differential expression of metabolism-related genes, including fatty acid, amino acid, CoA, and ATPase, between the high-risk and low-risk groups (**Figures 6B-E**). This suggested that MRGs might influence various metabolic pathways, thereby affecting the energy metabolism and biosynthesis of tumor cells, promoting the progression of BC.

### 3.7 Immunity analysis and immunotherapy response prediction

**Figure 7:**
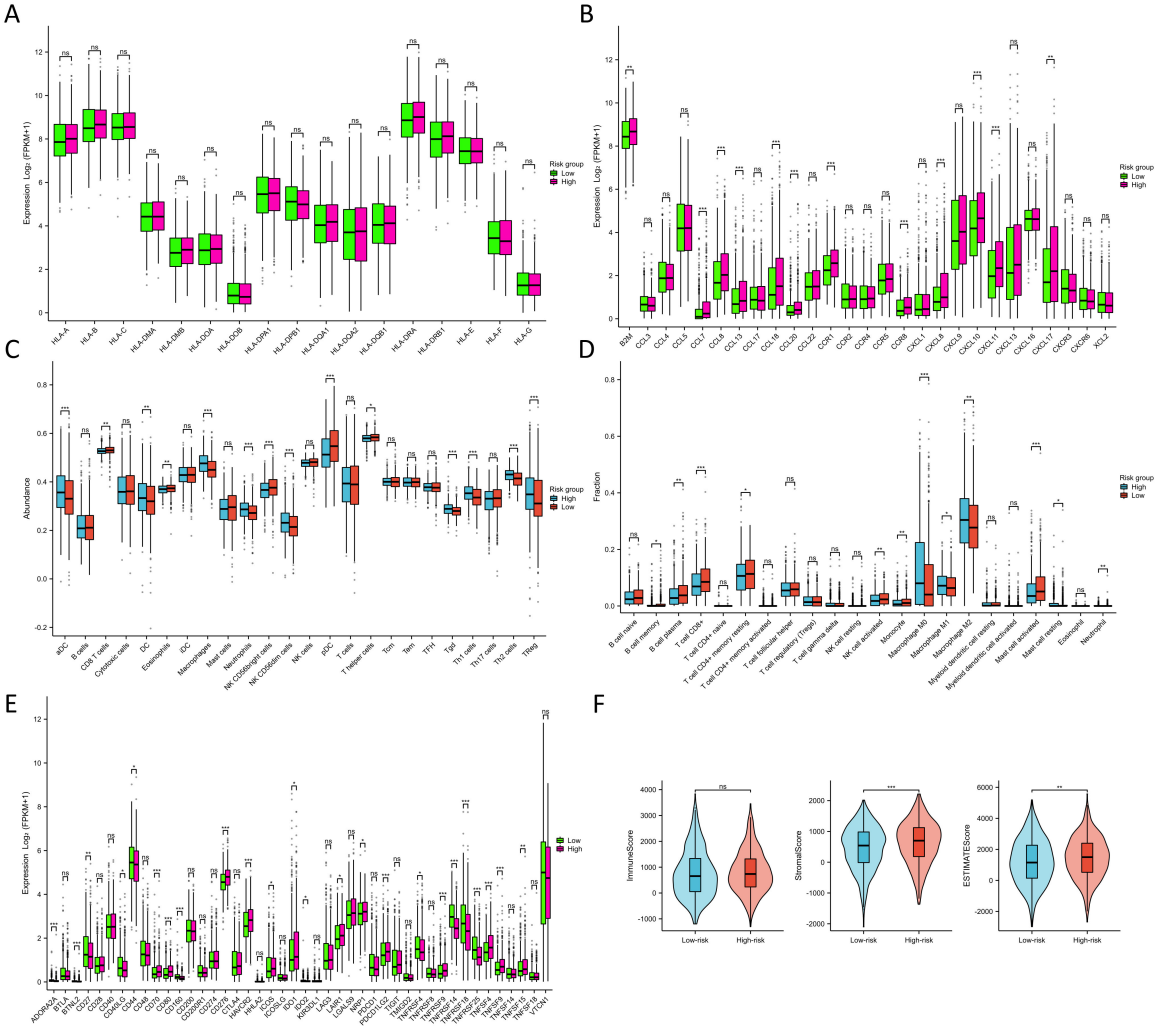
Immune characteristics between two risk groups. **(A)** Differences in MHC molecules between two risk groups. **(B)** Differences in chemokines and receptors between two risk groups. **(C)** Immune cell abundance between two risk groups calculated by the ssGSEA algorithm. **(D)** Immune cell infiltration between two risk groups calculated by the CIBERSORT algorithm. **(E)** Differences in ICG expression between two risk groups. **(F)** Differences in immune, stromal, and ESTIMATE scores between two risk groups. NS indicates no statistical difference, *P < 0.05, **P < 0.01, ***P < 0.001.

Throughout the course of tumorigenesis, mitochondrial metabolism can exert influence on immune cells within the tumor microenvironment (TME) (24). Therefore, we analyzed the immune characteristics in the TME between two risk groups. Interestingly, our analysis revealed no significant differences in the constituents of MHC-I and MHC-II between the two risk groups, as illustrated in **Figure 7A**. This observation suggests that MRGs might not influence the capability of antigen presentation. Notably, in the high-risk group, we observed a pronounced up-regulation of various chemokines and their receptors (**Figure 7B**), including CCL7, CCL8, CCL13, CCL18, CCL20, CCR1, CCR8, CXCL8, CXCL10, and CXCL11 (p<0.001). This up-regulation indicated a potential enhancement in the recruitment of anti-tumor immune cells within the TME as the patient risk score increased. However, the ssGSEA algorithm-based calculation of immune cell abundance revealed a significantly higher presence of most immune cells in the low-risk group. This included activated dendritic cells (aDCs), macrophages, neutrophils, natural killer (NK) CD56 dim cells, T helper 1 (Th1) cells, gamma delta T cells (Tgd), Th2 cells, and regulatory T cells (Tregs) (p<0.001) (**Figure 7C**). In addition, differences in the abundance of immune cells were observed in the high- and low-expression groups of 9 MRGs (**Supplementary Figure S7**). The CIBERSORT algorithm was also utilized to evaluate the infiltration levels of 22 distinct immune cell populations (**Figure 7D**). Our research has revealed a substantial enrichment of CD8+ T cells and active mast cells in the high-risk group (p<0.001). Moreover, M0, M1, and M2 macrophages were found to be notably enriched in the low-risk group (p<0.05). Further analyzing the expression of various immune cell signatures expression indicated that the majority of CD8+ T cell signatures were up-regulated in the high-risk group, corroborating our findings. Details of various immune cell signatures expression in both risk groups are presented in **Supplementary Figure S8**. Currently, immune checkpoint pathways and genes have been identified as promising avenues in cancer immunotherapy (25). We therefore evaluated the levels of 44 ICGs between two risk groups. Our findings indicated that the expression of ADORA2A, BTNL2, CD160, TNFRSF14, TNFRSF18, and TNFRSF25 were significantly elevated in the low-risk group, while in the high-risk group, CD70, CD276, HAVCR2, PDCD1LG2, and TNFSF9 were considerably higher (p<0.001) (**Figure 7E**). Furthermore, we observed that the stromal score and ESTIMATE score of the high-risk group were higher than those of the low-risk group (p<0.01) (**Figure 7F**). It has been established that a higher Stromal score may correlate with lower levels of T cell co-inhibitory/stimulatory molecules and angiogenesis markers (26). This could potentially contribute to the poorer prognosis of BC patients in the high-risk group. Our findings revealed distinct immune landscapes in the TME between the two risk groups, which may be one of the factors influencing the differences in prognosis between these groups, warranting further validation.

**Figure 8:**
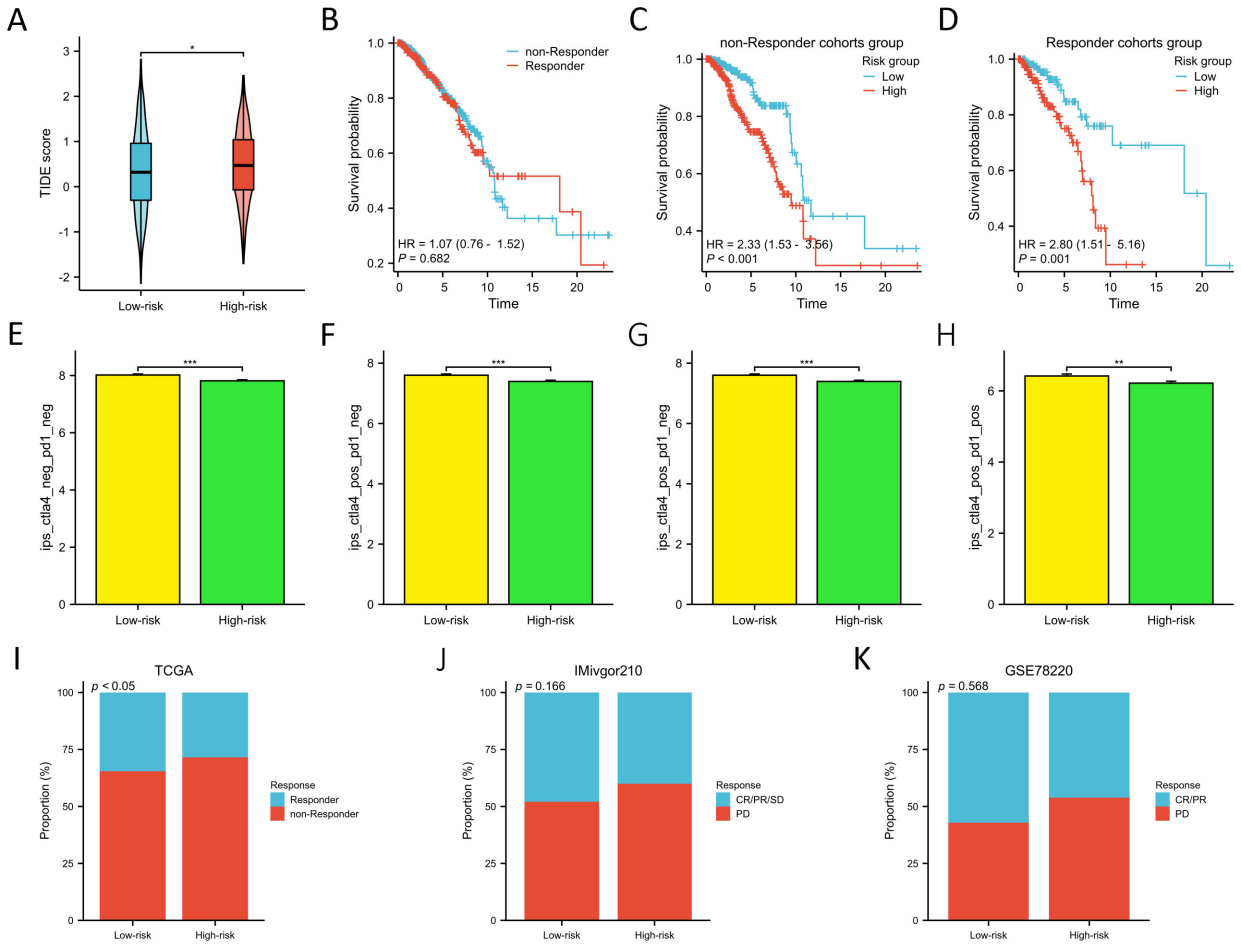
Prediction of immunotherapy response between two risk groups. **(A)** Differences in TIDE score between two risk groups. **(B)** KM survival curves in OS of the non-responder and responder cohorts. **(C, D)** KM survival curves in OS between two risk groups in the non-responder **(C)** and responder cohorts **(D)**. **(E–H)**. Differences of the IPS between two risk groups in both CTLA- and PD1-negative **(E)**, CTLA4-positive and PD1-negative **(F)**, CTLA4- negative and PD1- positive **(G)**, and both CTLA4- and PD1-positive cohorts **(H)**. **(I–K)** Proportion of the BC patients who non-respond and respond to immunotherapy in TCGA **(I)**, IMivgor210 **(J)**, and GSE78220 cohorts **(K)**. *P < 0.05, **P < 0.01, ***P < 0.001.

We employed the TIDE score to evaluate the predictive prowess of our risk model in forecasting the outcomes of immunotherapy in BC patients within the TCGA database. As depicted in **Figure 8A**, patients with a high risk score exhibited a significantly higher TIDE score compared to those with a low risk score (p<0.05), suggesting that patients with a low risk score might derive greater benefits from immunotherapy. KM survival analysis indicated no significant difference in OS between the non-responder and responder cohorts (**Figure 8B**). However, in both the non-responder and responder groups, individuals in the low-risk groups demonstrated improved prognosis (p<0.01) (**Figures 8C, D**). Furthermore, we employed the IPS algorithm to predict responses to anti-CTLA4 and anti-PD-1 therapies for each BC patient from TCGA. The results revealed that the low-risk group exhibited higher IPS in any CTLA4 and PD-L1 stratification (p<0.01) (**Figures 8E-H**), suggesting that BC patients with a low-risk score might be more responsive to immune checkpoint inhibitors. We further validated the predictive ability of the prognostic model for immunotherapy responses using two cohorts: anti-PDL1 in the IMvigor210 cohort and anti-PD1 in the GSE78220 cohort. Aligning with our findings from the TCGA training cohort (**Figure 8I**), patients in the low-risk groups in both the IMvigor210 and GSE78220 cohorts exhibited a higher response proportion compared to those in the high-risk groups (**Figures 8J, K**), although statistical significance was not reached. These results collectively demonstrate the superior predictive performance of the prognosis model in assessing the effectiveness of immunotherapy, indicating that patients with lower risk score were more likely to derive benefits from immunotherapy.

### 3.8 ITH analysis

ITH is one of the mechanisms leading to drug resistance in treatment, therefore, it is a significant challenge in clinical practice. To explore the relationship between the risk score and ITH of BC, we calculated ITH scores for each sample and divided them into ITH-low and ITH-high groups for further differential gene expression analysis between them (**Supplementary Table S11**). We identified the top 20 genes exhibiting differential expression related to ITH for further investigation of their associations with risk groups. Notably, these 20 ITH-differentially-expressed genes demonstrated lower expression in the ITH-high group (p<0.001) as shown in **Supplementary Figure S9A**. Furthermore, a majority of these genes also exhibited reduced expression in the high-risk group (**Supplementary Figure S9B**). Considering the low expression of these genes in both high-risk and ITH-high groups, we hypothesized a positive correlation between elevated ITH and increased risk scores, suggesting that high ITH may contribute to adverse prognoses. Subsequent analyses revealed that the ITH score was higher in the high-risk group (p<0.05) (**Supplementary Figure S9C**), and a positive correlation was observed between the ITH score and risk score (R=0.062, p=0.045) (**Supplementary Figure S9D**). Additionally, the prognosis for the ITH-high group was notably poorer compared to the ITH-low group (p<0.05) (**Supplementary Figure S9E**), reinforcing our hypothesis that high ITH likely serves as a critical factor influencing poor prognosis in BC. Moreover, within both ITH-low and ITH-high cohorts, patients in the high-risk groups exhibited significantly worse prognoses (p<0.01) (**Supplementary Figures S9F-G**).

### 3.9 Mutation, TMB, MSI, cancer stemness, and single cell analysis

Somatic mutations have significant impacts on the occurrence, development, and treatment of BC. Through in-depth research on these mutations, scientists can gain a better understanding of the molecular mechanisms of BC, laying the foundation for personalized treatment and the development of new therapies (27, 28). We conducted a comprehensive mapping of somatic mutation characteristics across two distinct risk groups, employing waterfall plots for visualization. The analysis for the high-risk group, as depicted in **Supplementary Figure S10A**, identified TP53 as the predominant mutation gene. In the low-risk group, PIK3CA emerged as the most frequently mutated gene, as shown in **Supplementary Figure S10D**. Notably, TP53, PIK3CA, and TTN were identified as common genes with high mutation frequencies in both groups. An examination of mutation statuses in both high- (**Supplementary Figure S10B**) and low-risk (**Supplementary Figure S10E**) groups revealed that single nucleotide variations (SNV) predominated as the most common variation type, with missense mutations being the most frequent variation classification in both cohorts. Comprehensive details regarding the base mutations, the proportion of base transitions (Ti) and transversions (Tv), along with a percentage breakdown of base mutations across all samples within both high- and low-risk groups, are illustrated in **Supplementary Figures S10C** and **S10F**, respectively.

Both TMB and MSI have been identified as promising predictive biomarkers for immunotherapy in cancer treatment (29). Additionally, cancer stemness not only correlates with tumor cell characteristics but also intertwines complexly with tumor development, prognosis, and immune infiltration (30, 31). In our analysis, we noted elevated TMB and mRNAsi score within the high-risk group (p<0.05) (**Supplementary Figure S11A**). There were significant positive correlations between the risk score and TMB (R=0.0198, p<0.001), as well as mRNAsi score (R=0.142, p<0.001) (**Supplementary Figure S11B**). Nonetheless, no noteworthy relationship was discernible between the MSI score and risk score (**Supplementary Figure S11A-B**). Despite patients in the low-risk group might derive greater benefits from immunotherapy, the high-risk group patients exhibited higher TMB levels, suggesting an enhanced capability for generating novel tumor antigens. This observation underscores the potential efficacy of immunotherapy in the high-risk group, meriting further exploration in clinical settings. Survival analysis revealed that TMB, MSI and mRNAsi scores were not associated with prognosis (**Supplementary Figure S11C**). Furthermore, within each subgroup, OS for high-risk patients was notably shorter compared to that of low-risk patients (p<0.05) (**Supplementary Figure S11D-E**).

We performed single-cell analysis using the BRCA_GSE148673 dataset from the TISCH database to examine the expression patterns of 9 prognostic NRGs in the tumor microenvironment-associated cells of BC. The annotation of cell types is displayed in **Supplementary Figures S12A** and **S12B**, encompassing 4 immune cell types, 4 stromal cell types, and malignant cells. The GSE148673 dataset comprises 28 distinct cell populations (**Supplementary Figure S12C**). **Supplementary Figures S12D** and **S12E** present the number of different cell types and the proportions of each cell type in various patients, respectively. Additionally, **Supplementary Figure S12F** illustrates the percentages and expressions of 9 prognostic MRGs. Among these MRGs, namely DCTPP1, KMO, NME3, CCR7, ISOC2, and COMTD1, their expressions were observed across multiple immune cell types. Specifically, DCTPP1, NME3, and ISOC2 were predominantly expressed in CD8T, CD4Tconv, and Mono/Macro cells. KMO exhibited a primary expression in Mono/Macro cells, while CCR7 and COMTD1 were primarily detected in CD8T and CD4Tconv cells. Notably, DCTPP1, NME3, and ISOC2 were also found to be expressed in stromal cells. Conversely, FEZ1, STAR, and ESR2 displayed minimal expression within the immune microenvironment.

### 3.10 Drug sensitivity analysis

To assess the predictive capacity of the risk score for drug treatment efficacy in BC patients, we calculated the IC50 values for 235 drugs and evaluated their correlation with the risk score, detailed in **Supplementary Table S12**. We pinpointed the top 10 drugs whose IC50 values demonstrated a negative correlation with the risk score and another 10 with a positive correlation. Detailed information about these drugs is presented in **Supplementary Tables S13, S14**, respectively. The top 10 drugs whose IC50 negatively correlated with the risk score were FTI-277, AKT inhibitor VIII, Thapsigargin, RO-3306, JNK-9L, AMG-706, TW 37, CH5424802, Docetaxel, and MS-275, all of which demonstrated significantly higher IC50 values in the low-risk group (p<0.001) (**Supplementary Figure S13A**). The top 10 drugs with IC50 values positively correlated with the risk score were TL-2-105, LAQ824, Belinostat, Navitoclax, I-BET-762, Dabrafenib, PHA-665752, AR-42, Linsitinib, and Phenformin. With the exception of AR-42, an increase in the IC50 of these drugs was observed in the high-risk group (p<0.05) (**Supplementary Figure S13C**). The risk score demonstrated potential discriminatory power in distinguishing between high-response and low-response groups for most drugs with negative IC50 correlations (**Supplementary Figure S13B**). However, this discriminatory power of the risk score was less evident in drugs with positive IC50 correlations to the risk score, as shown in **Supplementary Figure S13D**.

### 3.11 Establishment and evaluation of metastatic model

**Figure 9:**
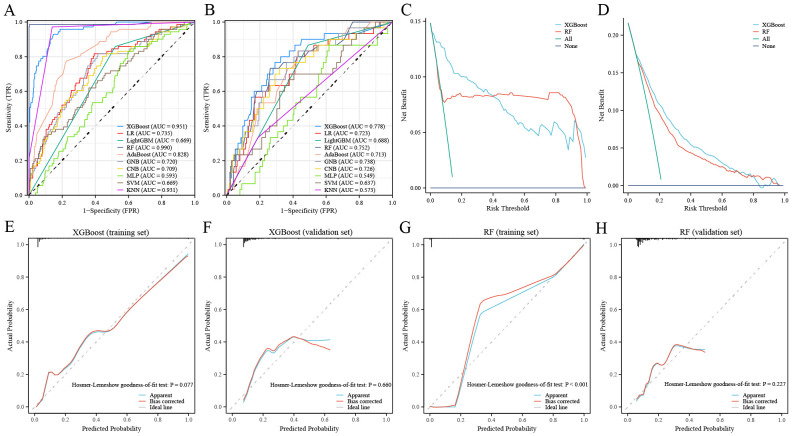
Performance of the distant metastasis machine-learning models. **(A, B)** ROC curves for the training set **(A)** and the validation set **(B)**. **(C, D)** Decision curves for the training set **(C)** and the validation set **(D)**. **(E, F)** Calibration curves of the XGBoost model in the training set **(E)** and the validation set **(F)**. **(G, H)** Calibration curves of RF model in the training set **(G)** and the validation set **(H)**.

We used the GSE102484 dataset, which included 101 distant metastasis and 582 non-metastasis BC samples, for the construction of the model for predicting distant metastasis. The results of the differential gene expression analysis between distant metastasis and non-metastasis groups are shown in **Supplementary Table S15**. The Boruta algorithm was used to determine the importance of the features of the metastatic models, and the results showed that PDK4, NRF1, DCAF8, CHPT1, MARS2, and NAMPT were included in subsequent construction of the models (**Supplementary Figure S14**). Detailed information on 6 metastasis-related MRGs are shown in **Table 3**. Compared with the other models, the XGBoost model not only demonstrated an excellent AUC of 0.951 in the training set but also achieved the highest AUC of 0.778 in the validation set (**Figure 9A**). While the RF model showed an outstanding AUC of 0.990 in the training set, it performed inferiorly compared to the XGBoost model in the validation set (**Figure 9B**). DCA was employed to determine the clinical utility of the models. The results indicated that both XGBoost and RF models had good net benefit in predicting metastasis in the training set, with XGBoost showing higher net benefit than RF in the validation set, thereby demonstrating greater clinical utility (**Figures 9C, D**). To further evaluate the probability prediction accuracy of the models, the calibration curves showed that the calibration of the RF model was inferior to that of the XGBoost model in both the training and validation sets (**Figures 9E-H**). **Table 4** shows the performance of the 10 machine-learning models in the training and validation sets. The XGBoost model in the training set showed an outstanding accuracy of 0.842, sensitivity of 0.859, specificity of 0.839, PPV of 0.506, NPV of 0.968, and F1 score of 0.628. Moreover, the XGBoost model in the validation set also possessed an accuracy of 0.737, sensitivity of 0.767, specificity of 0.709, PPV of 0.318, NPV of 0.935, and F1 score of 0.450. Therefore, in terms of comprehensive model evaluation, XGBoost was the optimal model for predicting metastasis in BC patients.

**Table 3:** The information of 6 metastasis-related MRGs.

Gene	Full name	Location	Function of the encoded protein
PDK4	Pyruvate dehydrogenase kinase 4	7q21.3	PDK4 protein plays a crucial role in the pathway of pyruvate metabolism by inhibiting the activity of pyruvate dehydrogenase through phosphorylation. This inhibition prevents the conversion of pyruvate to acetyl-CoA, thereby influencing the progression of the tricarboxylic acid cycle.
NRF1	Nuclear respiratory factor 1	7q32.2	NRF1 protein is a transcription factor associated with the cellular nuclear respiratory chain. It plays a role in regulating and coordinating mitochondrial biosynthesis, the expression of respiratory chain genes, and overall cellular energy regulation.
DCAF8	DDB1 and CUL4 associated factor 8	1q23.2	DCAF8 protein interacts with the CUL4-DDB1 E3 ligase macromolecular complex, playing a crucial role in biological processes such as cell cycle regulation and DNA damage response.
CHPT1	Choline phosphotransferase 1	12q23.2	CHPT1 is involved in the phosphatidylcholine biosynthetic process and platelet-activating factor biosynthetic process.
MARS2	Methionyl-tRNA synthetase 2	2q33.1	MARS2 protein is a methionyl-tRNA synthetase, primarily responsible for catalyzing the binding of methionine to its corresponding tRNA, forming aminoacyl-tRNA, and participating in the process of mitochondrial protein synthesis within the mitochondria.
NAMPT	Nicotinamide phosphoribosyltransferase	7q22.3	NAMPT protein catalyzes the condensation of nicotinamide with 5-phosphoribosyl-1-pyrophosphate to yield nicotinamide mononucleotide, a step in the biosynthesis of nicotinamide adenine dinucleotide.

**Table 4:** Performance of machine-learning metastatic models in training and validation sets.

Sets	Models	AUC	Accuracy	Sensitivity	Specificity	PPV	NPV	F1‐score
Training set	XGBoost	0.951	0.842	0.859	0.839	0.506	0.968	0.628
	LR	0.735	0.647	0.778	0.626	0.263	0.938	0.393
	LightGBM	0.669	0.835	0.805	0.593	0.131	0.878	0.205
	RF	0.990	0.998	1.000	1.000	1.000	0.997	0.993
	AdaBoost	0.828	0.776	0.772	0.770	0.376	0.946	0.505
	GNB	0.720	0.627	0.812	0.598	0.257	0.944	0.390
	CNB	0.709	0.592	0.802	0.558	0.237	0.938	0.366
	MLP	0.593	0.502	0.667	0.475	0.122	0.854	0.197
	SVM	0.669	0.639	0.723	0.627	0.275	0.930	0.380
	KNN	0.931	0.886	0.978	0.862	0.973	0.885	0.983
Validation set	XGBoost	0.778	0.737	0.767	0.709	0.318	0.935	0.450
	LR	0.723	0.605	0.567	0.829	0.226	0.920	0.323
	LightGBM	0.688	0.854	0.867	0.509	0.104	0.854	0.175
	RF	0.752	0.780	0.800	0.611	0.174	0.857	0.286
	AdaBoost	0.713	0.683	0.833	0.571	0.254	0.910	0.389
	GNB	0.738	0.615	0.667	0.743	0.247	0.944	0.361
	CNB	0.726	0.580	0.733	0.680	0.225	0.932	0.345
	MLP	0.549	0.190	0.933	0.171	0.153	0.975	0.263
	SVM	0.637	0.566	0.667	0.629	0.208	0.913	0.317
	KNN	0.573	0.844	0.333	0.811	0.250	0.856	0.286

### 3.12 Evaluation and interpretability of the XGBoost model

The confusion matrix visualized the performance of the XGBoost classifiers in the training and validation sets (**Supplementary Figure S15A, B**). The SHAP analysis provided an explanation of how the XGBoost model predicted distant metastasis and calculated the importance of features. **Supplementary Figure S15C** shows the SHAP values for each feature at different levels. As the feature values increase, the redder color is, and vice versa, the bluer color is. In addition, we ranked the features of the model (**Supplementary Figure S15D**). The higher feature ranking indicated that the feature is more important, which means that the feature contributes more to the model. Overall, the XGBoost model places the most importance on NAMPT, CHPT1, and MARS2. Furthermore, we presented two representative samples to elucidate the interpretability of the XGBoost model. One exemplifies a non-metastasis BC patient, characterized by a low SHAP prediction score of 0.23 (**Supplementary Figure S15E**), while the other metastasis BC patient exhibits a higher SHAP score of 0.66 (**Supplementary Figure S15F**).

## 4 Discussion

BC is the most widespread malignant neoplasm among women worldwide, with its prevalence intricately linked to a myriad of factors (1, 32). As such, the exploration of the complex mechanisms underpinning the development of BC and the creation of clinical prediction models for BC patients have become critical frontiers in BC research. This endeavor is crucial for guiding the development of efficacious treatment strategies for affected individuals. As the center of cellular energy metabolism, mitochondrial dysfunction is not only closely related to the occurrence and progression of cancer (7, 8) but also associated with drug resistance and the maintenance of tumor stem cells (9, 10). The relationship between mitochondria and BC is a significant area of research, and recent studies have revealed the intricate interactions between them. For example, through *in vitro* experiments and cancer cell transplantation experiments, research has found that inducing the expression of LACTB protein in BC cells can inhibit cell proliferation and differentiation (33). Additionally, burgeoning evidence indicates that MRGs are associated with the prognosis of BC patients, exemplified by genes such as SIRT5 and VDAC1 (12, 34). Additionally, MRGs have been linked to the metastasis of BC (35). Nonetheless, research investigating the relationship between MRGs and both the prognosis and metastasis of BC remains nascent. Reports of clinical prediction models being developed for BC patients based on MRGs are scant.

Currently, a substantial corpus of research has delved into the correlations between MRGs and cancer prognosis, with various models being formulated to predict outcomes (13, 36, 37). In this study, we have successfully constructed a prognostic model that forecasts OS in BC patients, employing 9 MRGs: DCTPP1, FEZ1, KMO, NME3, CCR7, ISOC2, STAR, COMTD1, and ESR2. Patients in the high-risk group exhibited a significantly poorer prognosis compared to their low-risk counterparts. The AUC of the time-dependent ROC curves for predicting 1-, 3-, and 5-year OS in the TCGA training cohort surpassed 0.7, affirming the model’s excellent predictive accuracy. This finding was further validated through both external and internal validation cohorts. Previously, Weixu et al. explored the significance of MRGs in BC, proposing a prognostic model based on 4 MRGs (38). In comparison, we included a greater number of MRGs in our prognostic analysis, which yielded a model with enhanced predictive precision. In addition, we constructed a nomogram combining the model’s risk score and the patient’s clinical characteristics, further confirming the accuracy and reliability of the model. Research indicates that DCTPP1 is up-regulated in BC, and high expression of DCTPP1 in BC is associated with poor prognosis, suggesting that DCTPP1 might play a significant role in the development of BC (39). Conversely, the body of research exploring the association between FEZ1 and BC remains sparse. Nevertheless, extant studies illustrate that introducing FEZ1 into FEZ1-deficient cancer cells significantly curtails tumorigenesis and reduces cellular proliferation, culminating in an accumulation of cells in the late S-G2/M phase of the cell cycle (40). Additionally, our findings indicate a notable down-expression of FEZ1 in BC tissues. Given FEZ1’s role as a tumor suppressor gene, its absence or reduced expression appears to significantly contribute to the advancement of BC. KMO is a key enzyme in the tryptophan metabolic pathway, primarily expressed at higher levels in triple-negative breast cancer (TNBC), and promotes the migration and invasion of TNBC cells, which might be independent of its enzymatic activity but through an β-catenin pathway (41). Current research on the correlation between NME3 and BC is limited, but studies suggest that it may play the role of a tumor suppressor gene (42). Our current findings indicate that patients with high expression of NME3 seem to have a better prognosis, although this is not statistically significant, further confirming its tumor-suppressing function. However, the anti-cancer mechanisms of NME3 in BC require further experimental exploration. The link between CCR7 and BC primarily manifests in its role in BC metastasis, notably influencing the migration and dissemination of tumor cells to the lymphatic system. Targeting the CCR7 axis may offer therapeutic avenues to mitigate BC spread (43, 44). Current knowledge regarding the involvement of ISOC2, STAR, and COMTD1 in BC is lacking. Our study finds their expression correlates with the infiltration of various immune cells, suggesting a potential role in the tumor immune microenvironment of BC. However, elucidating their precise functions in BC onset and progression requires further investigation. It is established that ESR2 exhibits reduced expression in BC, and its low expression levels are linked to enhanced OS rates, potentially through modulation of immune responses (45). Although our current research also confirms the low expression of ESR2 in BC, no significant differences in OS were observed, indicating the need for more extensive experiments to decode ESR2’s role in BC.

In this study, the outcomes of the functional enrichment analysis revealed that MRGs were primarily associated with the progression of metabolism involved in the progression of BC, mainly nucleotide, fatty acid, and amino acid metabolism. Therefore, we analyzed the differences in metabolism-related genes expression between two risk groups. Aberrations in nucleotide metabolism extend beyond tumor proliferation to encompass diverse oncogenic behaviors such as immune evasion, metastasis, and therapy resistance (46). Our findings indicate a downregulation of enzymes catalyzing nucleotide hydrolysis in the high-risk group, suggesting that the impact of MRGs on nucleotide metabolism predominantly manifests through the modulation of nucleotide hydrolase expression and activity, thereby influencing the proliferation of BC cells. Additionally, we noted variations in the expression of other metabolism-related genes between the two risk groups, highlighting a multifaceted and profound interconnection between energy metabolism and cancer development. Tumor cells alter the flux through various metabolic pathways to meet their increased demands for bioenergy and biosynthesis, while simultaneously mitigating oxidative stress, thus promoting the proliferation and survival of tumor cells. Furthermore, fibroblasts and immune cells within the TME also regulate tumor progression through their metabolism (24, 47). In summary, energy metabolism plays a crucial role in the development of BC, and metabolic pathways regulated by mitochondrial genes hold promise as new therapeutic targets for BC.

Mitochondrial metabolism exerts a notable influence on immune cells within the TME, prompting us to examine the immune characteristics of two distinct risk groups. Chemokines and their receptors exert pro- or anti-tumoral role in cancer progression through their involvement in leukocyte recruitment, angiogenesis, cancer cell proliferation, and metastasis (48, 49). Our analysis revealed that patients in the high-risk group typically exhibit elevated levels of chemokine and receptor expression, alongside increased infiltration of CD8+ T cells and reduced presence of various immunosuppressive cell types, such as Tregs, M0, and M2 macrophages. Research indicates that chemokines and their receptors within the TME play a crucial role in regulating the migration and function of CD8+ T cells, such as CXCL9 and CXCL10 (50). Consequently, we hypothesize that the pronounced infiltration of CD8+ T cells in patients of the high-risk group is linked to the activity of chemokines. Furthermore, M2 macrophages and Treg cells are known to create an immunological barrier that impedes the anti-tumor immune response mediated by CD8+ T cells (51). Intriguingly, a similar pattern was observed among patients in the low-risk group, rather than those in the high-risk group, suggesting that in the latter, the recruitment of anti-tumor immune cells and the suppression of immunosuppressive cells may be consequences of the body’s defensive response to the tumor, rather than the causes. Nevertheless, we noted elevated infiltration levels of anti-tumor immune cells in the low-risk group, including aDCs, NK CD56 dim cells, and Th1 cells, which may contribute to a more favorable prognosis for these patients. The activation of DCs is essential for the successful presentation of cancer antigens to T cells, which results in a specific immune attack on tumor cells (52). NK cells, particularly those characterized by the expression of CD56 dim, play a significant role in the body’s immune response against cancers. These cells are a subset of NK cells that are known for their potent cytotoxic activity against tumor cells (53). Recent studies have highlighted the increasing importance of eliciting a Th1 response in cancer immunotherapy, which can kill tumor cells indirectly by activating cytotoxic T lymphocytes and antigen-presenting cells, and directly by releasing cytokines that activate death receptors on the surface of tumor cells (54). Furthermore, we observed that the Stromal score and ESTIMATE score of the high-risk group were higher than those of the low-risk group. It has been reported that higher stromal score and lower immune score may correlate with higher levels of T cell co-inhibitory/stimulatory molecules and angiogenesis markers (26), which may contribute to the poorer prognosis of BC patients in the high-risk group. Nonetheless, no differences in immune scores were observed between the two groups. Given these findings, we propose that the TME in both groups contributes to both cancer promotion and tumor suppression. The immune characteristics of the two groups likely not be the primary cause of the differences in prognosis. Thus, a deeper understanding of the TME’s complexities is essential for the development of more efficacious immunotherapeutic approaches.

The TIDE score is used to predict the tumor’s immune escape capability by comprehensively evaluating the activity of two main mechanisms in the tumor: T cell dysfunction and tumor immune exclusion (55). The IPS algorithm is a measure for evaluating the likelihood of a patient’s response to ICIs, such as PD-1/PD-L1 and CTLA-4 inhibitors (18). Patients in the low-risk group exhibited lower TIDE scores and higher IPS scores, regardless of their PD-1 or CTLA-4 status. This indicates that patients at lower risk are more likely to benefit from immunotherapy. This finding implies that stratifying patients by risk can more accurately predict who will respond better to immunotherapy, thereby supporting personalized immunotherapy decisions. Moreover, analyses of real-world cohorts of patients receiving immunotherapy, specifically the IMvigor210 and GSE78220 cohorts, have also confirmed the model’s predictive performance in evaluating responsiveness to immunotherapy. These studies underscore the utility of the model in a clinical setting, providing a more accurate prediction of patient outcomes following immunotherapy treatment. Additionally, through drug sensitivity analysis, we found that patients in the high-risk group are more sensitive to treatments with drugs such as FTI-277. FTI-277 is a compound that exhibits significant anti-proliferative effects on BC cells, particularly showing a strong anti-proliferative action on H-Ras-MCF10A and Hs578T BC cells expressing active H-Ras mutations (56). In contrast, patients in the low-risk group are more sensitive to drugs such as TL-2-105. Currently, there are no studies on the correlation between TL-2-105 and BC treatment, and more detailed information is needed to fully understand its mechanism of action and potential for clinical application. In summary, drug sensitivity analysis has revealed differences in sensitivity to specific drugs among patients in different risk groups, providing important information for the development of personalized treatment plans.

The association between ITH and cancer has emerged as a focal point in BC research recently. The high tumor heterogeneity associated with poor prognosis is also one of the main determinants of treatment resistance and failure (19). In this study, we observed a positive correlation between risk score and ITH score, and ITH-high was related to poor prognosis in BC patients. This relationship might explain the poorer prognosis and reduced responsiveness to immunotherapy observed in high-risk groups, thereby lending additional support to the interpretability of our model.

Machine-learning models demonstrate immense potential in clinical applications; however, successfully integrating these models into clinical practice requires overcoming challenges related to data quality, model interpretability, regulatory compliance, and ensuring fairness and ethics in their application (57). Distant metastasis in BC is a primary cause of poor prognosis and treatment failure in patients. At present, there are relatively few studies focused on predicting BC metastasis through model construction. Previously, one study selected target genes for distant metastasis of BC and constructed multiple 21-gene models for predicting distant metastasis using different machine-learning methods. Among these, the model based on the random forest algorithm performed the best in predicting the occurrence of distant metastasis (58). Mitochondria plays a crucial role in the metastasis of BC, largely due to their involvement in metabolic reprogramming and the regulation of cellular processes that promote cancer progression and resistance to treatment (35). Our study is pioneering in constructing a clinical prediction model for BC distant metastasis using six mitochondrial-related genes (MRGs): PDK4, NRF1, DCAF8, CHPT1, MARS2, and NAMPT. This represents one of the innovative aspects of our current research. PDK4 is a key enzyme involved in regulating glucose metabolism and mitochondrial respiration, which has been shown to be relatively overexpressed in BC and associated with poor prognosis (59). NRF1 is involved in mitochondrial energy metabolism and is highly expressed in BC tissues, which upregulates the expression of ROS scavenging enzymes SOX2 and GPX1, allowing tumor cells to maintain low levels of ROC, thereby promoting tumor cell epithelial-mesenchymal transition (EMT) and metastasis (60). DCAF8, through its interaction with the CUL4-DDB1 E3 ubiquitin ligase complex, participates in the ubiquitination and subsequent proteasomal degradation of proteins, playing a crucial role in several biological processes closely related to the development and progression of cancer (61). CHPT1 is found to be significantly overexpressed in BC, mediating pivotal metabolic alterations within BC cells and contributing to the enhancement of the malignant phenotype and the proliferation of BC cells, and associated with the early metastasis of tamoxifen-resistant BC cells (62). No studies currently link MARS2 with BC, but in non-small cell lung cancer, MARS2 is known to impact glycolysis and cellular redox balance through its interaction with MCU, facilitating EMT and cancer progression (63). Research indicates that NAMPT accelerates the proliferation of BC cells by activating AKT and ERK1/2 pathways, NAMPT inhibitors hold promise as novel therapeutic approaches for BC treatment (64).

In the current study, the XGBoost model showed the best performance with an accuracy of 0.842, sensitivity of 0.859, specificity of 0.839, PPV of 0.506, NPV of 0.968, and F1 score of 0.628 in the training set. The establishment of a distant metastasis prediction model for BC through MRGs not only helps us understand the mechanisms of MRGs in BC metastasis but also provides a basis for personalized treatment of BC patients.

This study has developed predictive models for BC prognosis and distant metastasis by analyzing MRGs. These models exhibit high accuracy in forecasting OS and the likelihood of distant metastasis in BC patients. Against the backdrop of the increasing focus on personalized medicine, the MRGs identified in this research offer novel perspectives for the individualized treatment of BC. Specifically, the predictive models constructed herein can assist clinicians in estimating patient responses to chemotherapy or immunotherapy based on individualized risk scores, thereby guiding the selection of treatment strategies, optimizing therapeutic plans, and minimizing unnecessary side effects. Moreover, for high-risk patients, targeted interventions aimed at enhancing mitochondrial function may be considered as an adjunct to standard treatment protocols to improve therapeutic outcomes.

However, despite the significant contributions of this study, several limitations must be acknowledged. First, the retrospective design of this study inherently restricts the ability to establish causal relationships with certainty. Second, although the study utilized data from multiple databases, the potential lack of population diversity within these datasets may limit the generalizability of the models. Furthermore, while several MRGs associated with prognosis were identified, the precise biological functions and mechanisms of these genes in BC remain inadequately understood. Lastly, the drug sensitivity analyses conducted in this study were based on *in vitro* data, necessitating further validation through *in vivo* experiments and clinical trials to support the translation of these findings into clinical practice. Nonetheless, these findings lay the groundwork for future research. Further investigations are needed to elucidate the underlying mechanisms by which these MRGs influence BC progression and metastasis. A deeper understanding of these pathways could lead to the identification of new therapeutic targets, potentially resulting in the development of more effective treatments. Additionally, clinical trials are necessary to validate the clinical utility of the MRG-based models developed in this study, ensuring their reliability in guiding treatment decisions. Moreover, given that mitochondrial dysfunction is a hallmark of various cancer types, future research should explore the role of these MRGs in other malignancies, potentially contributing to the development of cross-cancer therapeutic strategies.

## 5 Conclusion

In summary, based on MRGs we successfully constructed a prognostic model to predict OS and immunotherapy responses for BC patients. Moreover, we established a model for predicting distant metastasis. The formulation of these dual clinical prediction models offers valuable insights for personalized and precise treatment strategies and heralds new avenues for investigating the pathogenesis of BC. Moving forward, it is crucial to further explore the roles of MRGs in BC.

## Data Availability

The raw data supporting the conclusions of this article will be made available by the authors, without undue reservation.

## Glossary

BC: breast cancer
OXPHOS: oxidative phosphorylation
ROS: reactive oxygen species
MRGs: mitochondrial-related genes
TCGA: The Cancer Genome Atlas
GEO: Gene Expression Omnibus
OS: overall survival
GO: Gene Ontology
KEGG: Kyoto Encyclopedia of Genes and Genomes
GSEA: Gene Set Enrichment Analysis
PPI: protein-protein interaction
MHC: major histocompatibility complex
ICGs: immune checkpoint genes
ssGSEA: single sample gene set enrichment
TIDE: Tumor Immune Dysfunction and Exclusion
IPS: Immunophenoscore
ITH: intratumor heterogeneity
TMB: tumor mutational burden
MSI: microsatellite instability
mRNAsi: mRNA expressionbased stemness index
IC50: 50% inhibitory concentration
GDSC: Genomics of Drug Sensitivity in Cancer
RF: random forest
XGBoost: extreme gradient boosting
LR: logistic regression
LightGBM: light gradient boosting machine
AdaBoost: adaptive boosting
GNB: gaussian naive bayes
CNB: complement naive bayes
MLP: multi-layer perceptron neural networks
SVM: support vector machine
KNN: k-nearest neighbors
AUC: area under the curve
PPV: positive predictive value
NPV: negative predictive value
DCA: Decision Curve Analysis
SHAP: SHapley Additive exPlanations
qRT-PCR: quantitative real-time polymerase chain reaction
IHC: immunohistochemistry
ER: estrogen receptor
PR: progesterone receptor
HER2: human epidermal growth factor receptor 2;
TME: tumor microenvironment
aDC: activated dendritic cells
NK: natural killer
Th1: T helper 1
Tgd: gamma delta T cells
Tregs: regulatory T cells;
SNV: single nucleotide variations
TNBC: triple-negative breast cancer
EMT: epithelial-mesenchymal transition
