# Supplementary material for: Mitochondrial-related genes as prognostic and metastatic markers in breast cancer: insights from comprehensive analysis and clinical models
Source: Front Immunol. 2024 Sep 24;15:1461489. doi: 10.3389/fimmu.2024.1461489 (PMC11458410; doi:10.3389/fimmu.2024.1461489)
Supplement: Supplementary file 1 [file DataSheet1.docx]

**
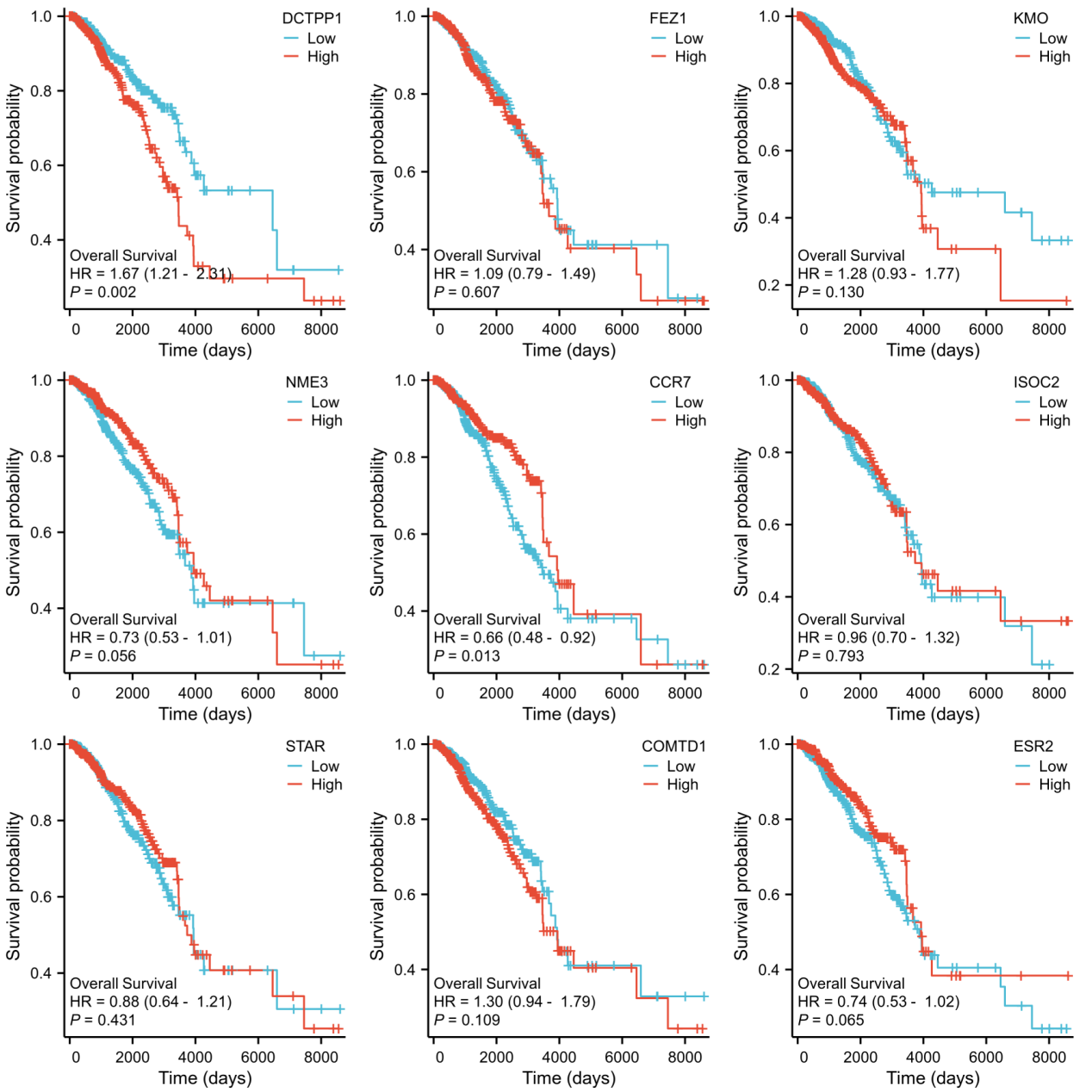
**

**Supplementary Figure S1.** KM survival analysis of 9 prognostic MRGs.

**
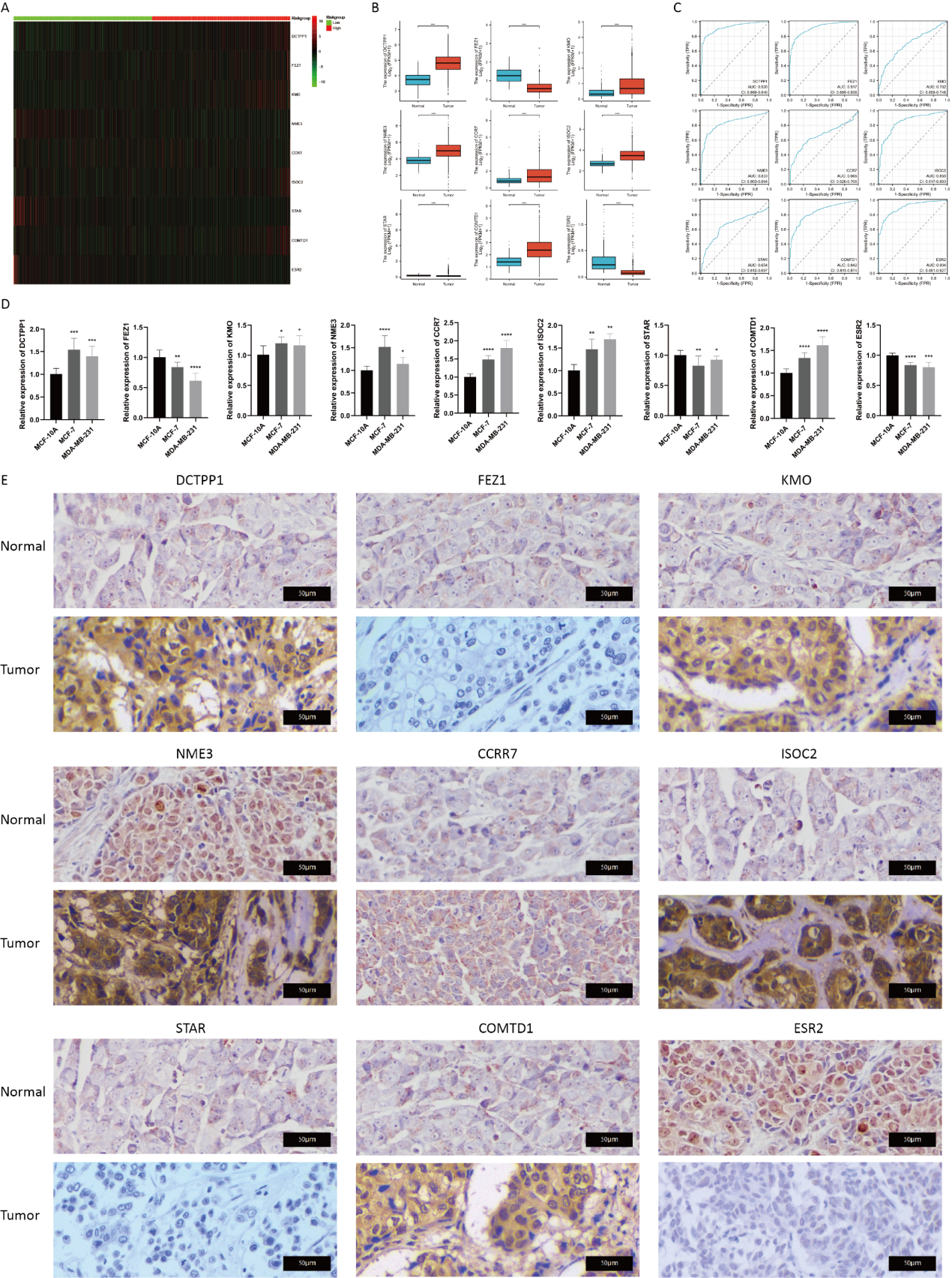
**

**Supplementary Figure S2.** Expression of 9 prognostic MRGs. (**A**) Heat map shows the expression of 9 MRGs between high- and low-risk groups. (**B**) Expression of 9 MRGs between normal and BC tissue groups. (**C**) ROC curves show the diagnostic performance of 9 MRGs. (**D**) QRT-PCR for validation of expression of 9 MRGs in cell lines. (**E**) Representative images of IHC for validation of protein expression of 9 MRGs BC tissues and their matched paracancerous tissues. Original magnifications 200×. *P < 0.05, **P < 0.01, ***P < 0.001, ****P < 0.0001.


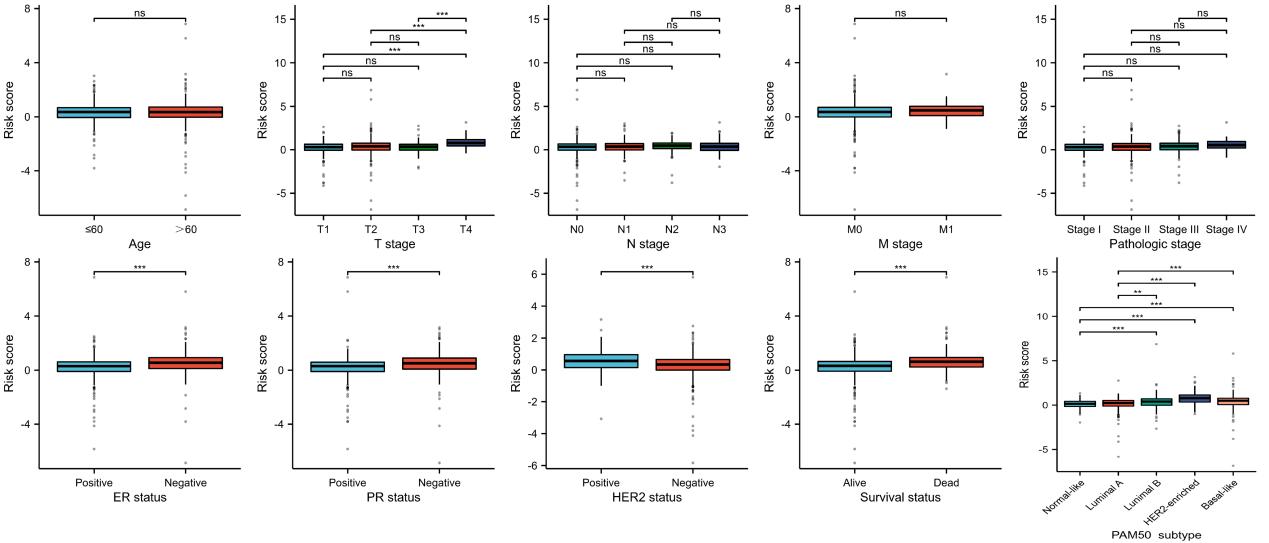


**Supplementary Figure S3.** Correlation between risk score and clinical characteristics. NS indicates no statistical difference, **P < 0.01, ***P < 0.001.

**
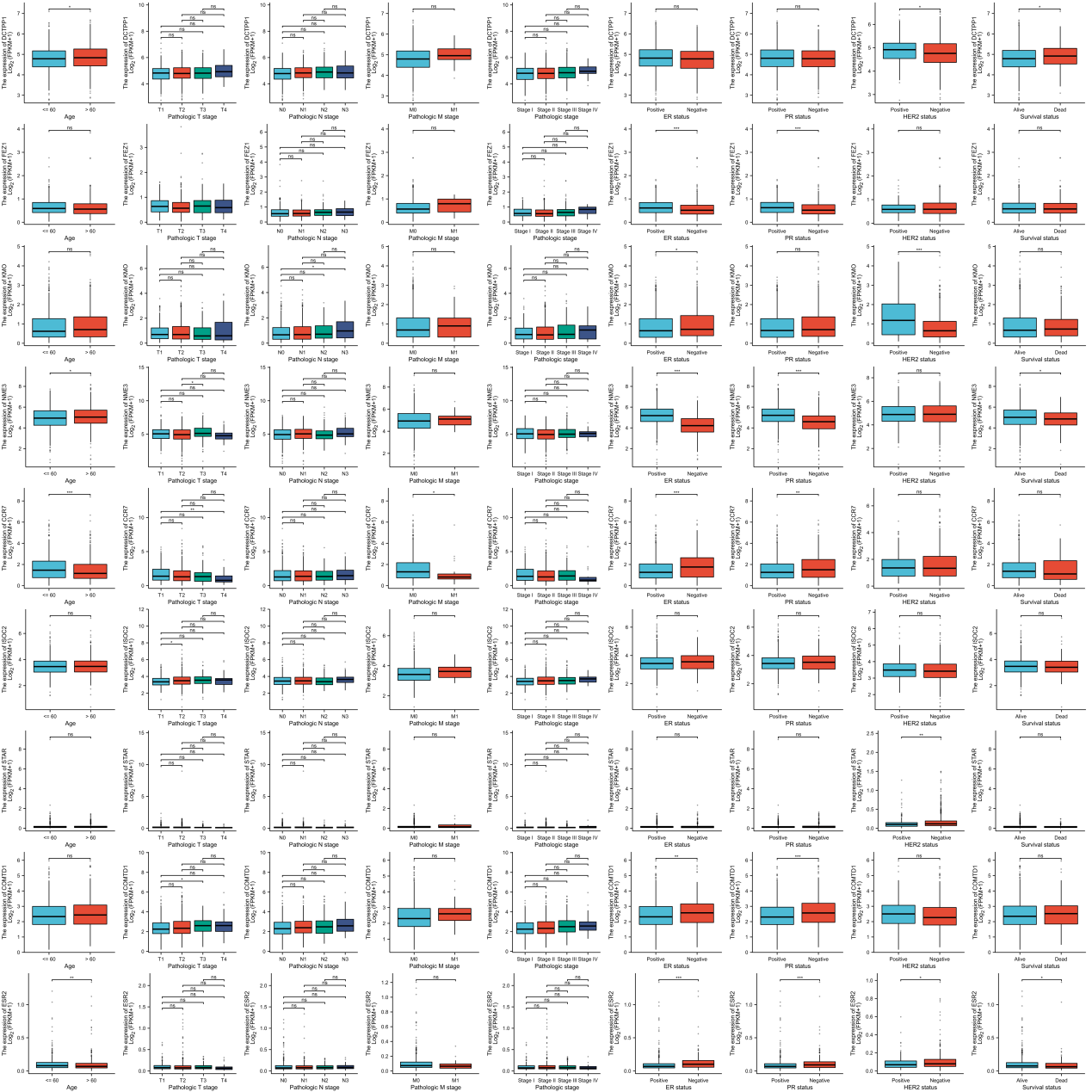
**

**Supplementary Figure S4.** Correlation between 9 prognostic MRGs and clinical characteristics. NS indicates no statistical difference, *P < 0.05, **P < 0.01, ***P < 0.001.


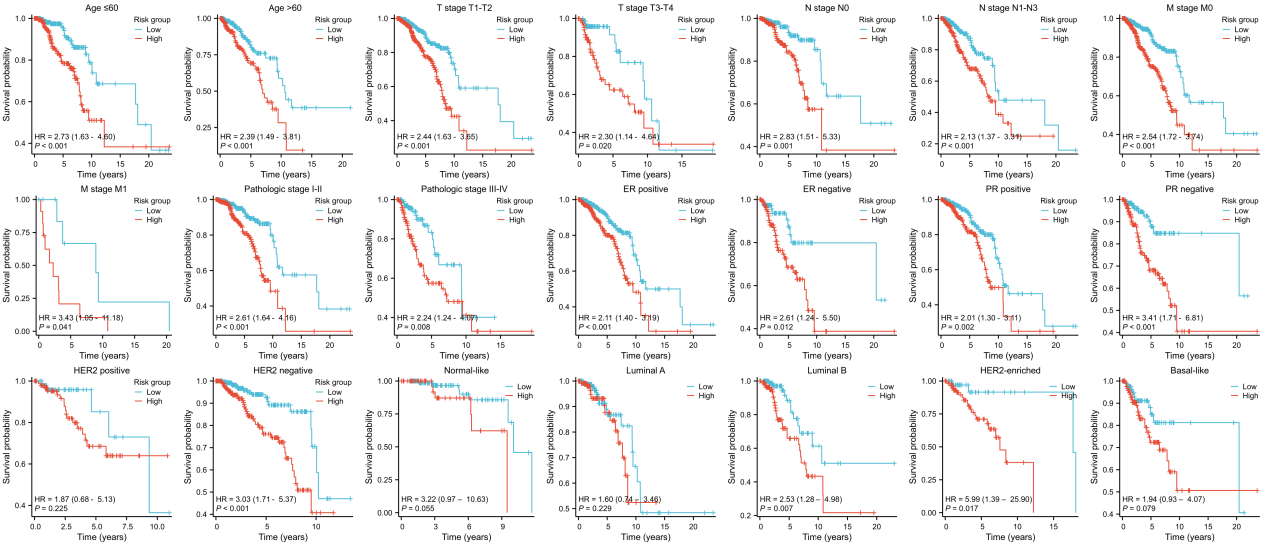


**Supplementary Figure S5.** KM survival analysis of the OS between two risk groups in various clinical subgroups.


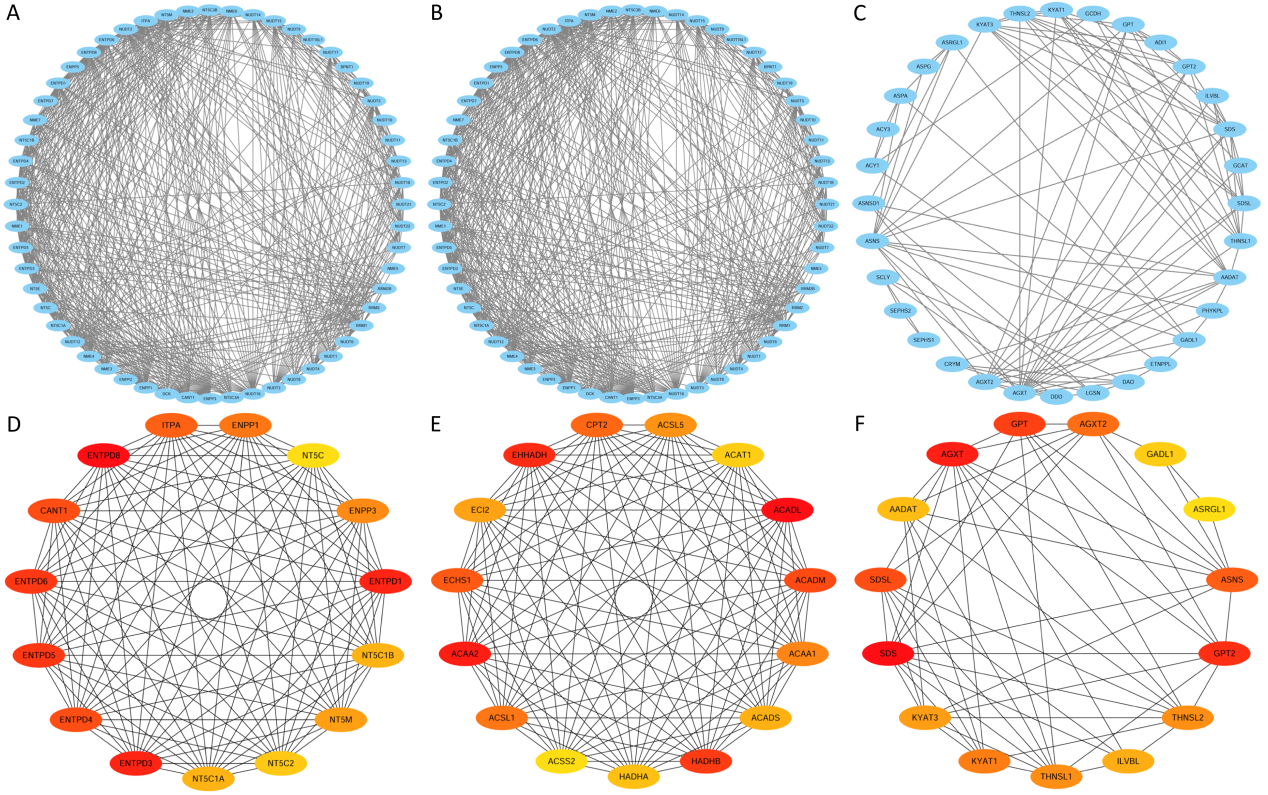


**Supplementary Figure S6.** PPI networks construction and hub genes selection for metabolism-related genes. (**A-C**) PPI networks of 63 nucleotide (A), 139 fatty acid (B) and 38 amino acid (C) metabolism-related genes. (**D-F**) 15 hub genes selection for further analysis based on PPI networks of nucleotide (D), fatty acid (E) and amino acid (F) metabolism-related genes.


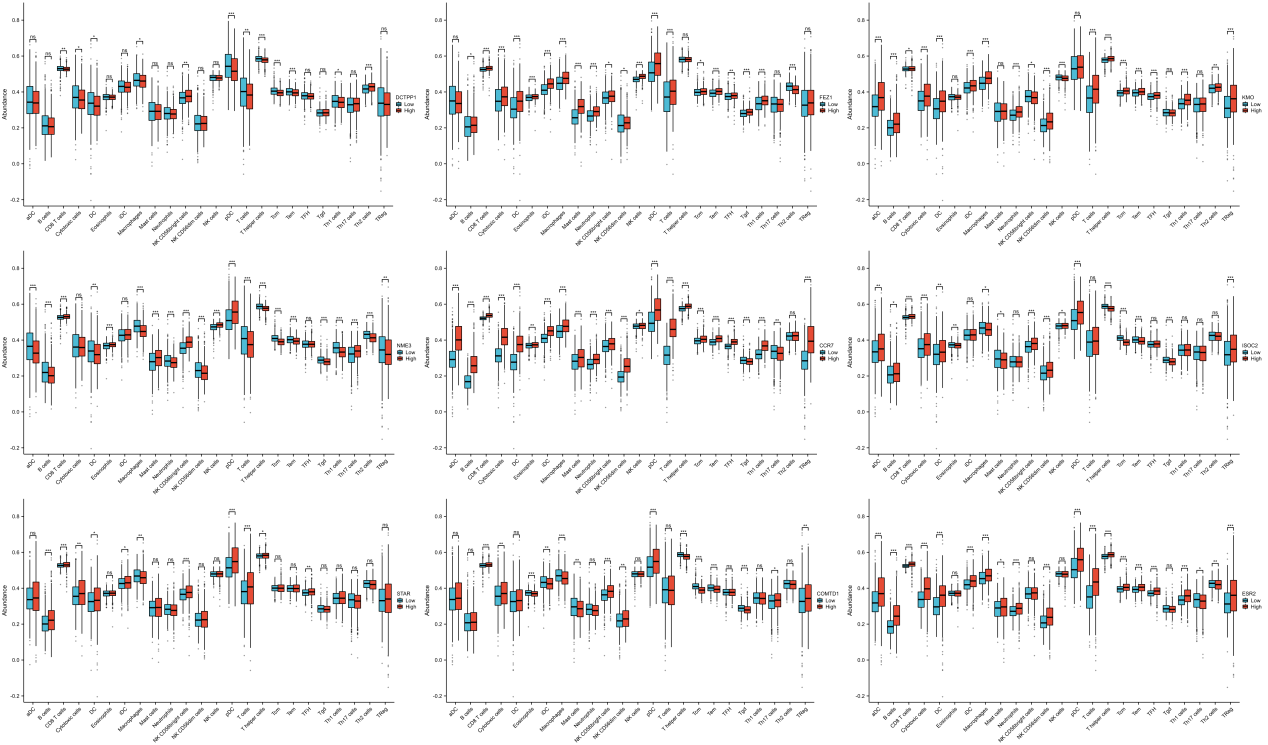


**Supplementary Figure S7.** Immune cell abundance in the high- and low-expression groups of 9 prognostic MRGs calculated by the ssGSEA algorithm. NS indicates no statistical difference, *P < 0.05, **P < 0.01, ***P < 0.001.


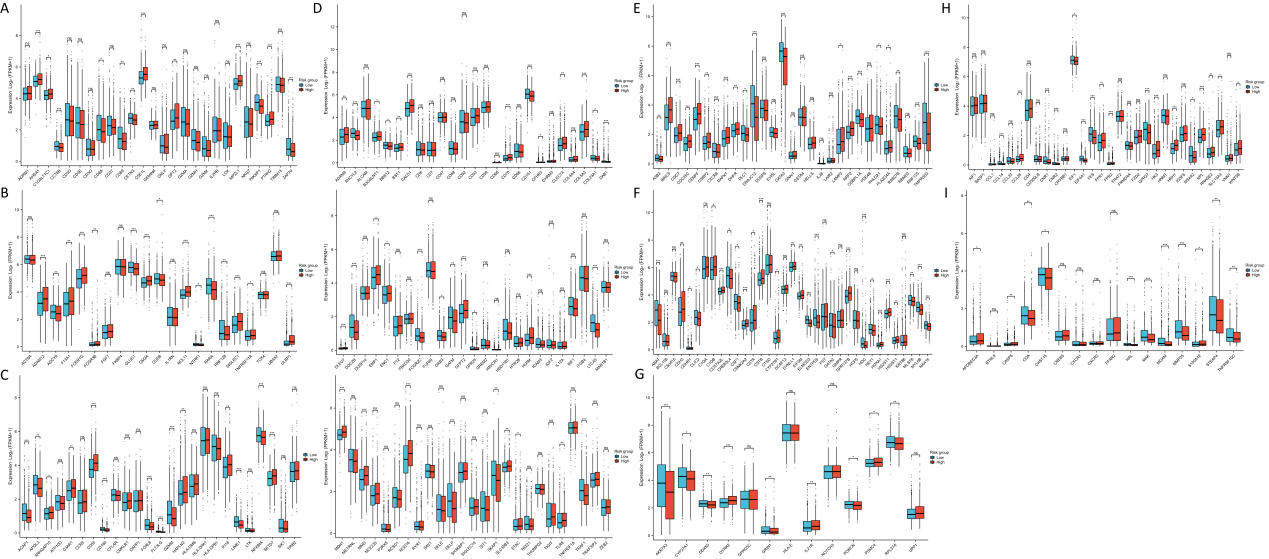


**Supplementary Figure S8.** Immune cell signatures expression of activated CD8+ T cell (A), central memory CD8+ T cell (B), effector memory CD8+ T cell (C), Th1 cells (D), Th2 cells (E), NK CD56 bright cells (F), NK CD56 dim cells (G), macrophage (H) and neutrophil (I) between two risk groups. NS indicates no statistical difference, *P < 0.05, **P < 0.01, ***P < 0.001.


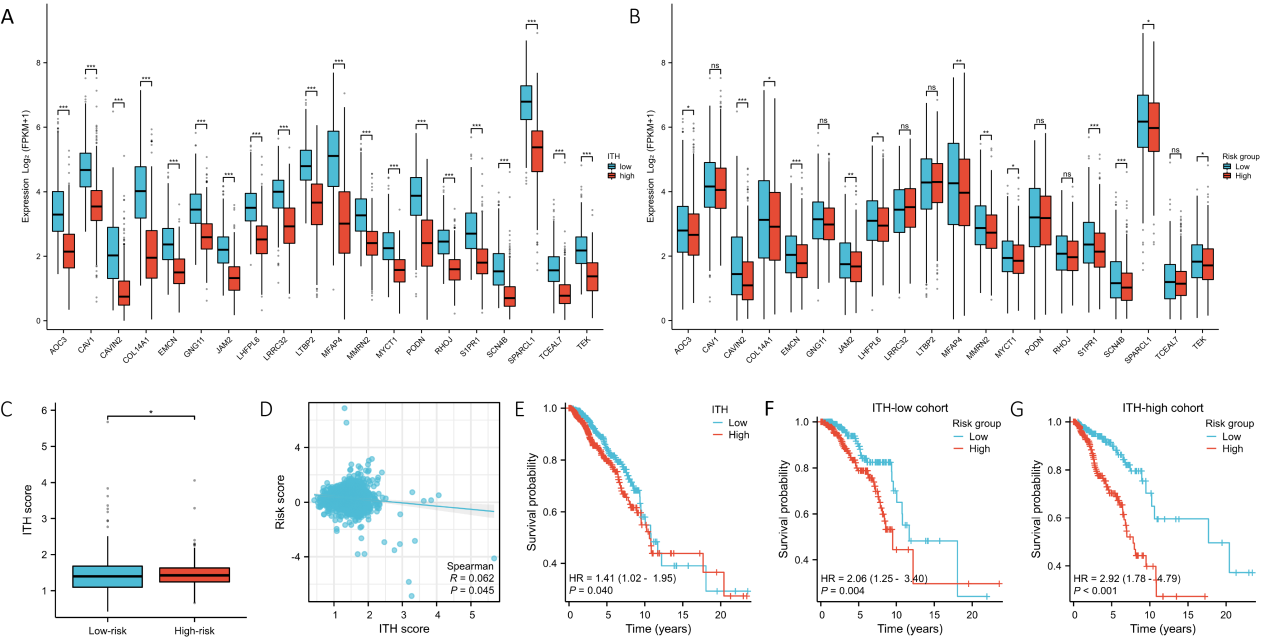


**Supplementary Figure S9.** ITH analysis between two risk groups. (**A**) The expression of the top 20 ITH-differentially-expressed genes in ITH-low and ITH-high cohorts. (**B**) The expression of IHT-differentially-expressed genes in low- and high-risk groups. (**C**) Differences in ITH score between two risk groups. (**D**) Correlation analysis between the ITH score and risk score. (**E**) KM survival curves in OS of the ITH-low and ITH-high cohorts. (**F, G**) KM survival curves in OS between two risk groups in the ITH-low (F) and ITH-high cohorts (G). NS indicates no statistical difference, *P < 0.05, **P < 0.01, ***P < 0.001.


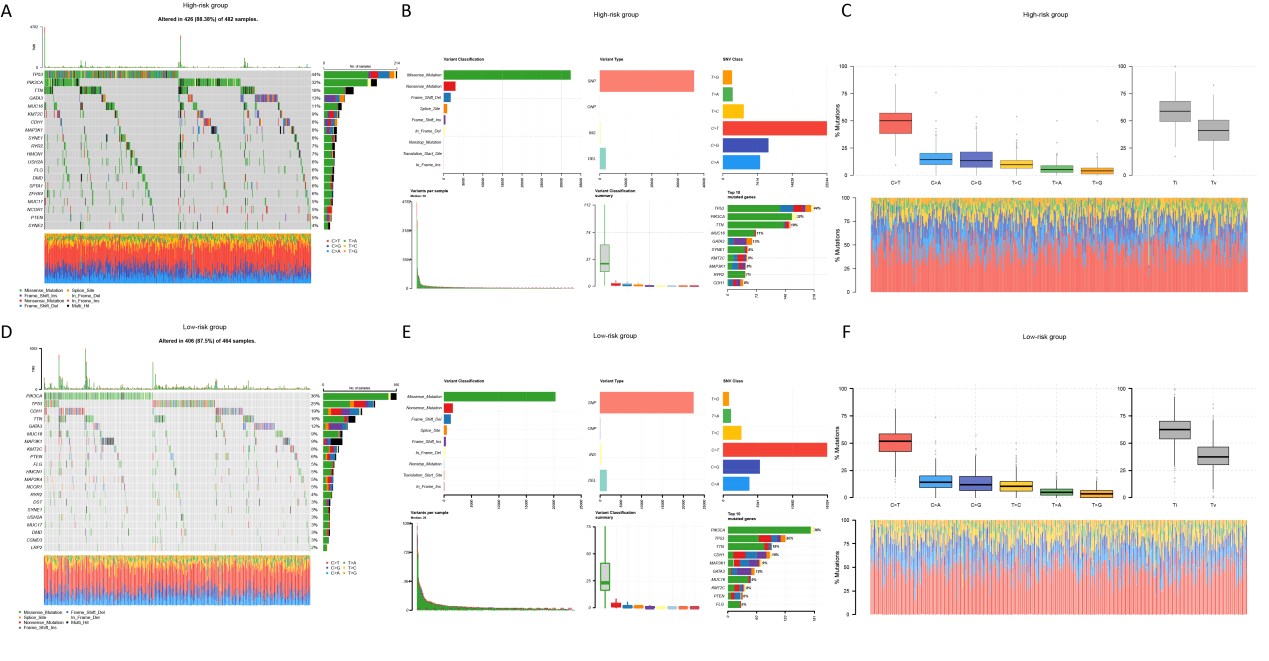


**Supplementary Figure S10**. Mutation analysis between two risk groups. (**A, D**) The waterfall plots of somatic mutation features established with high- (A) and low-risk (D) groups. (**B, E**) Mutation status statistics in high- (B) and low-risk (E) groups. (**C, F**) Statistics on base mutations, the proportion of base transitions (Ti) and transversions (Tv), and the percentage of base mutations across all samples in high- (C) and low-risk (F) groups.


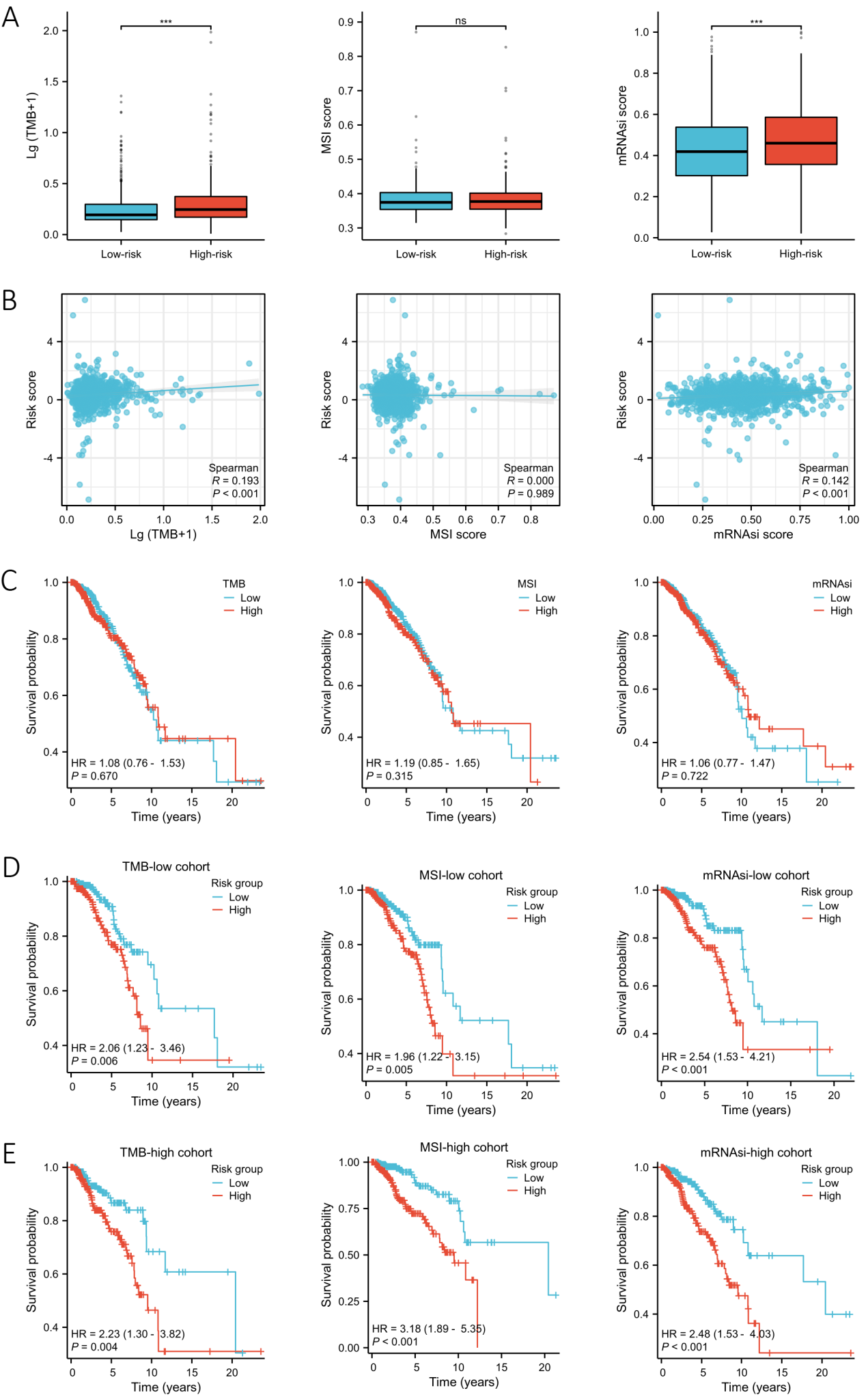


**Supplementary Figure S11.** TMB, MSI, and cancer stemness analysis between two risk groups. (**A**) Differences in TMB, MSI and mRNAsi scores between two risk groups. (**B**) Correlation analysis between the risk score and TMB, MSI and miRNAsi scores. (**C**) KM survival curves in OS between the TMB-low and TMB-high, MSI-low and MSI-high, and mRNAsi-low and mRNAsi-high cohorts. (**D**) KM survival curves in OS between two risk groups in the TMB-low, MSI-low, and mRNAsi-low cohorts. (**E**) KM survival curves in OS between two risk groups in the TMB-high, MSI-high, and mRNAsi-high cohorts. NS indicates no statistical difference, ***P < 0.001.


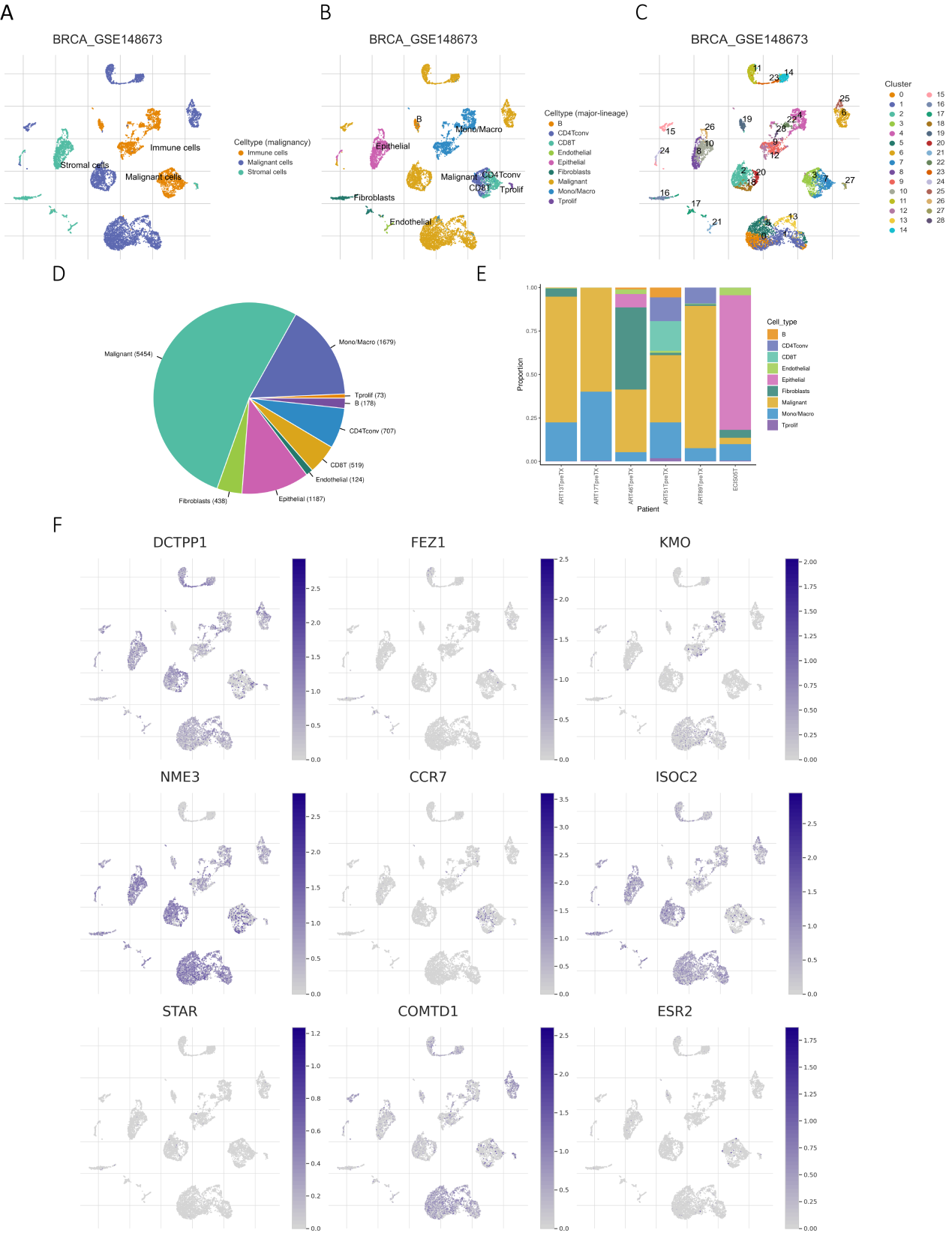


**Supplementary Figure S12.** Single cell analysis of MRGs expression in BC TME-associated cells via the GSE148673 dataset. (**A, B**) Annotation of the cell types. (**C**) Annotation of the clusters. (**D**) Number of various cell types. (**E**) Proportions of each cell types in different patients. (**F**) Percentages and expressions of 9 prognostic MRGs.


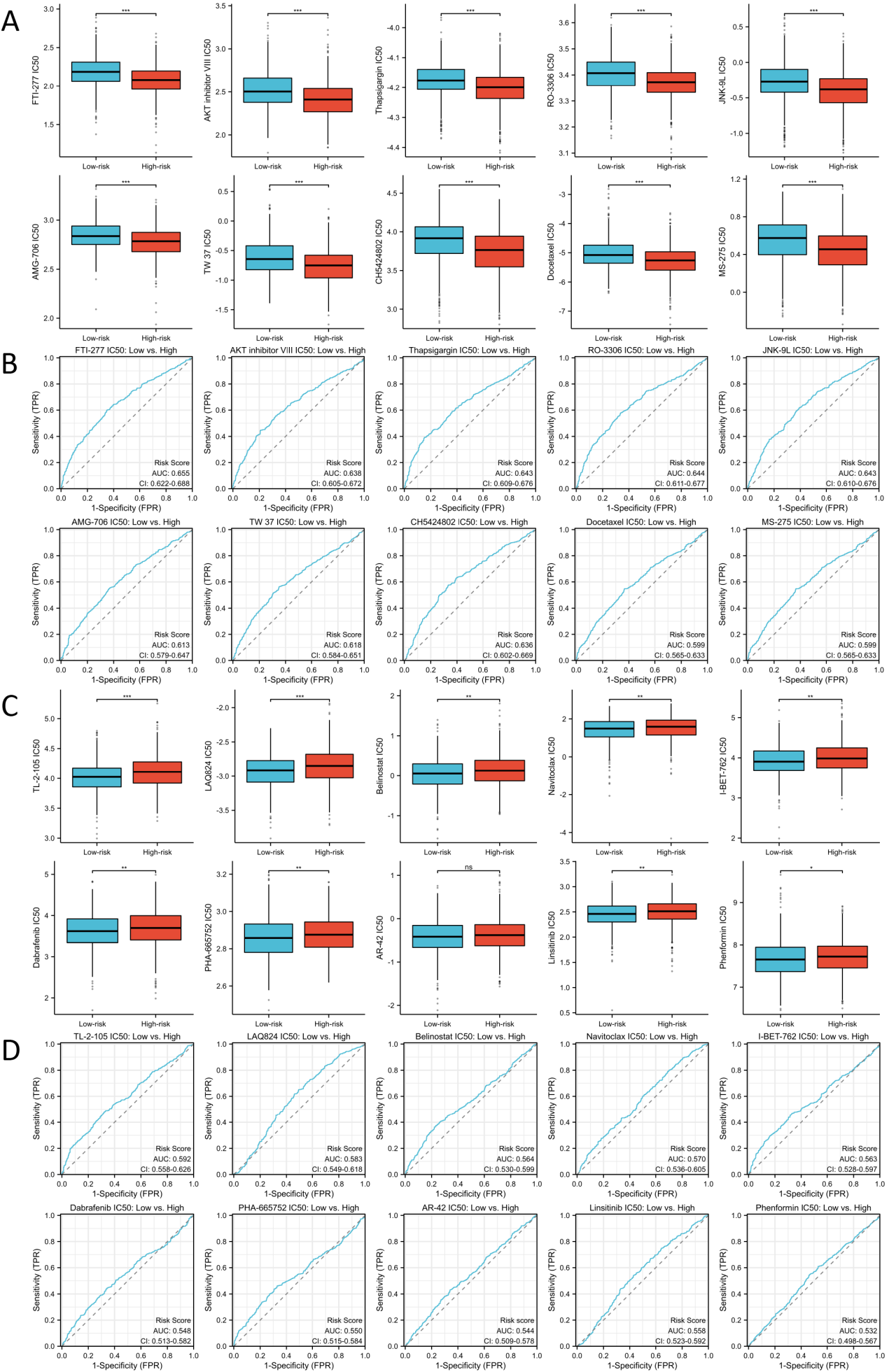


**Supplementary Figure S13.** Drug sensitivity analysis. (**A, C**) Differences in IC50 values of the top 10 drugs negatively (A) and positively (C) correlate with risk score in two risk groups. (**B, D**) ROC analysis to evaluate the discriminative power of the risk score in the high-response and low-response groups of the top 10 drugs negatively (B) and positively (D) correlate with risk score. NS indicates no statistical difference, *P < 0.05, **P < 0.01, ***P < 0.001.


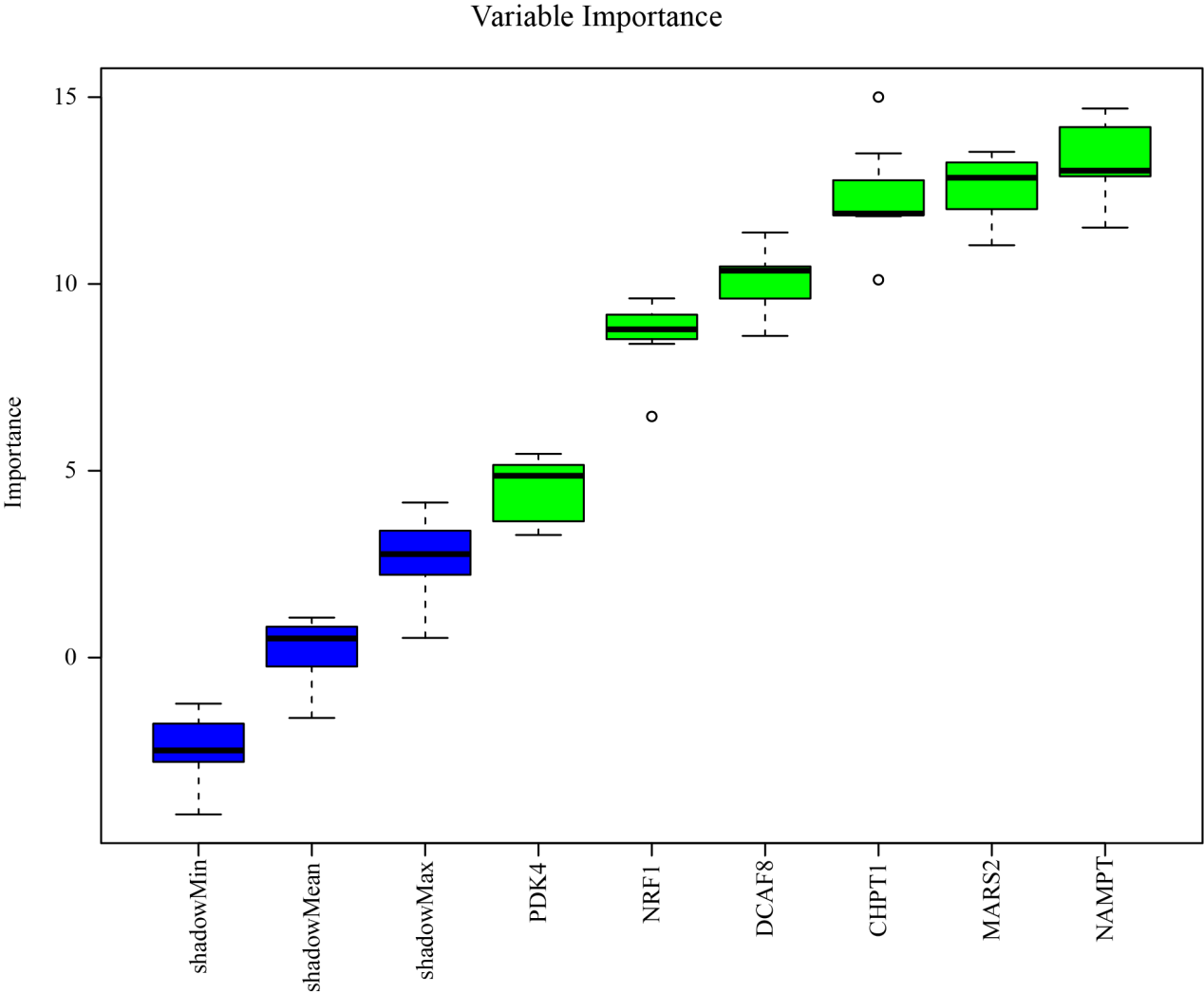


**Supplementary Figure S14.** The importance of the features of the metastatic model determined by the Boruta algorithm.


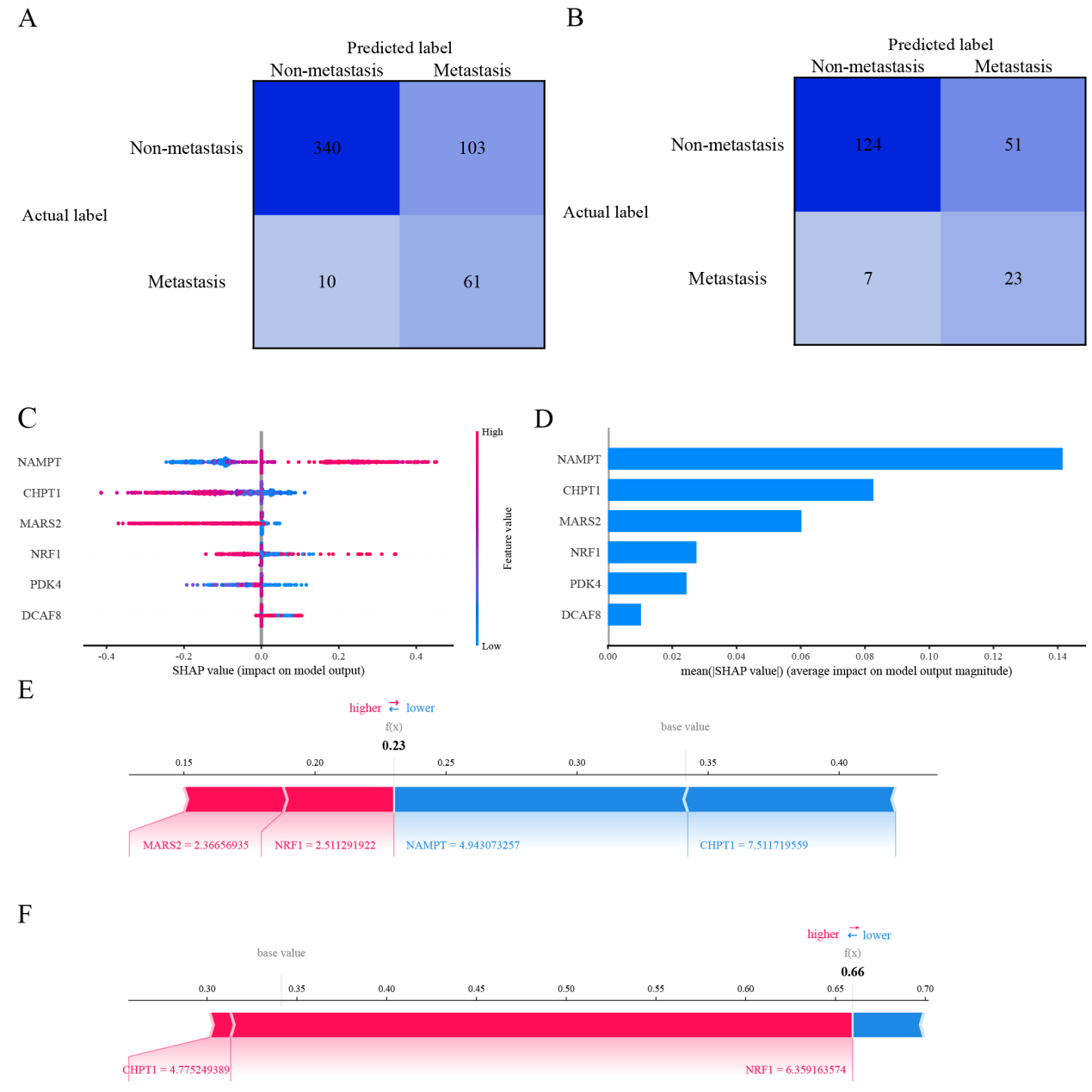


**Supplementary Figure S15.** Evaluation and interpretability of the XGBoost model. (**A, B**) Confusion matrix of the XGBoost model in the training set (A) and the validation set (B). (**C**) SHAP values for each feature at different levels in the XGBoost model. (**D**) Important features of the XGBoost model. (**E**) Interpretability of non-metastasis patients whose predictions were negative. The contributing variables are arranged in the horizontal line, sorted by the absolute value of their impact. The output value is the predicted risk of metastasis. (**F**) Interpretability of metastasis patients whose predictions were positive. The contributing variables are arranged in the horizontal line, sorted by the absolute value of their impact. The output value is the predicted risk of metastasis.
